# The Genetic Architecture of Methotrexate Toxicity Is Similar in *Drosophila melanogaster* and Humans

**DOI:** 10.1534/g3.113.006619

**Published:** 2013-08-01

**Authors:** Galina Kislukhin, Elizabeth G. King, Kelli N. Walters, Stuart J. Macdonald, Anthony D. Long

**Affiliations:** * Department of Ecology and Evolutionary Biology, University of California, Irvine, Irvine, California 92697-2525; † Department of Molecular Biosciences, University of Kansas, Lawrence, Kansas 66045

**Keywords:** *Drosophila* synthetic population resource, pharmacogenomics, methotrexate, quantitative trait loci, chemotoxicity

## Abstract

The severity of the toxic side effects of chemotherapy varies among patients, and much of this variation is likely genetically based. Here, we use the model system *Drosophila melanogaster* to genetically dissect the toxicity of methotrexate (MTX), a drug used primarily to treat childhood acute lymphoblastic leukemia and rheumatoid arthritis. We use the *Drosophila* Synthetic Population Resource, a panel of recombinant inbred lines derived from a multiparent advanced intercross, and quantify MTX toxicity as a reduction in female fecundity. We identify three quantitative trait loci (QTL) affecting MTX toxicity; two colocalize with the fly orthologs of human genes believed to mediate MTX toxicity and one is a novel MTX toxicity gene with a human ortholog. A fourth suggestive QTL spans a centromere. Local single-marker association scans of candidate gene exons fail to implicate amino acid variants as the causative single-nucleotide polymorphisms, and we therefore hypothesize the causative variation is regulatory. In addition, the effects at our mapped QTL do not conform to a simple biallelic pattern, suggesting multiple causative factors underlie the QTL mapping results. Consistent with this observation, no single single-nucleotide polymorphism located in or near a candidate gene can explain the QTL mapping signal. Overall, our results validate *D. melanogaster* as a model for uncovering the genetic basis of chemotoxicity and suggest the genetic basis of MTX toxicity is due to a handful of genes each harboring multiple segregating regulatory factors.

Methotrexate (MTX) is among the many chemotherapeutic medications prescribed to patients that have been shown to be very toxic (Wecker *et al.* 2010). MTX is an antifoltate drug used primarily to treat childhood acute lymphoblastic leukemia and rheumatoid arthritis. Its toxicity includes gastrointestinal symptoms such as nausea, vomiting, abdominal pain, and diarrhea; central nervous system toxicity; hepatitis with increased transaminase levels; serious or life-threatening skin reactions; and decrease in the activity of the patient’s immune system (http://www.nlm.nih.gov/medlineplus). Although MTX is effective in the treatment of some patients, for others, its toxicity is unbearable, and patients often are forced to change the dose or switch to a different drug (Hardman *et al.* 1996; Rothenberg *et al.* 2001); in the meantime, the disease progresses. This difference in a patient’s ability to tolerate such toxic medications has often been attributed to environmental factors such as diet, medical history, and age. However, these factors do not explain all of the underlying toxicity (Gajewski *et al.* 1989; Meirow and Nugent 2001; Rothenberg *et al.* 2001; Watters and McLeod 2003; Lee *et al.* 2005), and there is strong evidence that, alternatively, genes in concert with the environment govern toxicity (Cirulli and Goldstein 2010). Although it is desirable to study the underlying genetic architecture of MTX’s toxicity, doing so in humans is difficult. Chemotherapy dosing often is personalized, a patient can be taking several medications, and interindividual variation in environmental factors such as age, weight, and diet makes genetic characterization of toxicity challenging.

We have previously shown that there is a strong genetic component to MTX toxicity, as quantified by the reduction in fecundity after a 3-d MTX treatment regime, in female *Drosophila melanogaster* (Kislukhin *et al.*, 2012). In female *D. melanogaster* treated orally with MTX, the observed reduction in fecundity is proportional to drug concentration and treatment length and is therefore an objective and quantitative way to measure chemotoxicity. It is not surprising that MTX affects the reproductive ability of *D. melanogaster* because MTX competitively inhibits difolate reductase (DHFR). DHFR is needed for DNA synthesis during cell replication; therefore, MTX inhibits cell division required for reproduction of both humans and flies. With *D. melanogaster*, it is also possible to standardize the environment and hence maximize the power of an experiment to identify genetic factors underlying the toxicity phenotype. We have previously shown the reduction in fecundity after MTX exposure has a high heritability (99%; Kislukhin *et al.* 2012), suggesting it is possible to identify specific genes underlying the MTX toxicity in *D. melanogaster* and their human orthologs.

A powerful approach to identifying the genetic factors underlying complex traits is to use a collection of recombinant inbred lines (RILs) derived from a synthetic population or “collaborative cross” type base population (Churchill *et al.* 2004; MacDonald and Long 2007). Synthetic populations cross multiple inbred founder lines for multiple generations to create a population whose genomes are mosaics of the original founder lines’ genomes. Panels using a synthetic population approach are expected to perform particularly well when rare alleles of large effect underlie the trait of interest, for which there are an increasing number of examples (*e.g.*, Cohen *et al.* 2004; Ji *et al.* 2008; Stefansson *et al.* 2008; Helbig *et al.* 2009; Nejentsev *et al.* 2009; Pinto *et al.* 2010; Rivas *et al.* 2011; reviewed in Bansal *et al.* 2010). Recently, the *Drosophila* Synthetic Population Resource (DSPR) was created as a community resource for the genetic dissection of complex traits (http://FlyRILs.com; King *et al.* 2012a,b). This panel consists of more than 1700 RILs generated from two different 8-way, 50-generation synthetic populations. These RILs are a stable genetic reference panel that allows for high-powered and high-resolution quantitative trait loci (QTL) mapping in the *D. melanogaster* model system.

Here, we use the DSPR to genetically dissect MTX toxicity as measured by a reduction in fecundity in MTX exposed female flies. We successfully map three QTL influencing MTX toxicity. Two of the QTL are located in regions of the *Drosophila* genome harboring the fly orthologs of human genes believed to mediate MTX toxicity (either through their being in the biochemical pathway involved in processing MTX or their harboring SNP that have been shown to have clinical effects on toxicity). The third QTL was used to identify a novel candidate gene. We find little evidence that the underlying DNA polymorphisms explaining the QTL encode amino acid variants, instead we speculate the genetic variation to toxicity is regulatory in nature. This observation is apropos in light of the fact that human chemotoxicity pharmacogenomics has focused almost exclusively on amino acid encoding variants. Local association scans of candidate gene regions (using *in silico* derived genomes for the RILs) for toxicity suggest that QTL cannot be explained by a single genetic polymorphism. We argue that variation for toxicity may be due to a handful of genes each harboring several causative alleles.

## Materials and Methods

### Preliminary drug delivery and dose−response assay

We performed dosing experiments to determine the dose response effect of MTX. Eight doses (varying 3^6^-fold plus a placebo, no drug, control) of MTX were mixed with liquid food, spread onto a 1-cm × 2-cm piece of Whatman filter paper (cat. no. 3030 392), and placed into an agar plug at the bottom of a plastic exposure vial. Twelve 3-d-old male and female flies were placed in each of six exposure vials per dose, exposed to drug/food or placebo/food filter papers for 3 d, and then transferred to recovery vials for one day. After recovery, four females were chosen at random from each recovery vial and placed individually in separate “lay-out” vials, allowed to lay eggs for 3 d, and discarded (see Supporting Information, Table S1 for recovery and lay-out food composition). Two weeks after the mothers were discarded from the lay-out vials, the offspring were recovered and counted. We chose the final dose for the MTX toxicity phenotype experiment, based on a 50% fecundity decrease, as 110 μM.

### Mapping population

We used RILs from the DSPR (http://FlyRILs.com) to map QTL for MTX toxicity. The DSPR is a multifounder advanced intercross panel consisting of a set of more than 1700 RILs of *Drosophila melanogaster*. Two 8-way synthetic populations (pA and pB) were created from two independent sets of seven inbred founder lines (A1−A7 or B1−B7) with one additional line (AB8) shared by both populations. Each synthetic population was maintained as two independent replicate subpopulations (pA1 and pA2 or pB1 and pB2), kept at a large population size, and allowed to freely recombine for 50 generations. At generation 50, each subpopulation gave rise to ~500 RILs via 25 generations of full-sib mating. The genomes of the original 15 inbred founder lines have been completely resequenced, and the complete underlying founder haplotype structure of all RILs in the panel has been determined via a hidden Markov model (HMM). Complete details of the development of the DSPR, founder whole genome resequencing, and RIL genotyping are described in King *et al.* (2012a). The development of the HMM to infer the mosaic structure of the RILs and the power and mapping resolution of the DSPR for QTL mapping are described in King *et al.* (2012b). All of the raw genomic data and inferred founder genotype data are freely available at http://FlyRILs.org.

### The MTX toxicity phenotype

**Figure 1:**
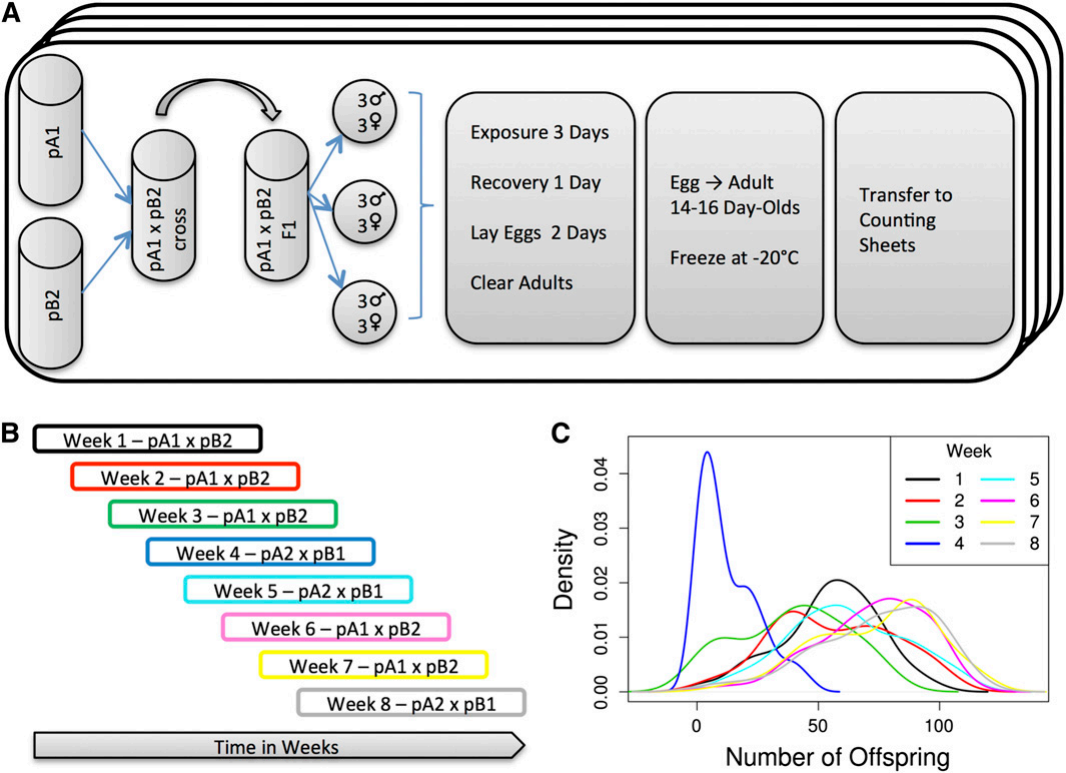
Experiment Design. (A) Each pair of RILs was crossed to produce the F1 hybrid. The hybrids were raised to adulthood, brother-sister mated, and placed into condo wells (three males and three females per well). Each condo contained four RIL crosses exposed to MTX simultaneously. After a 3-d exposure, each condo was recovered for 1 d, and the flies were allowed to lay eggs for 2 d after the recovery stage, after which the adult flies were cleared out of the condo. The eggs developed into adults for 14 d after the condos were cleared, frozen at −20°, and transferred onto adhesive sheets for counting. (B) The entire assay was split into eight blocks, with start dates on consecutive weeks. Each block took 8 wk to complete. F1 hybrids from populations A1×B2 were assayed over the first 3 wk, and any crosses that failed to complete the entire cycle were repeated in weeks 6 and 7. F1 hybrids from populations A2×B1 were assayed in weeks 4 and 5, and the crosses that failed in those two weeks were completed in week 8. (C) Density plot of the mean number of offspring produced by the three replicate females from each RIL. Different colors correspond to different weeks (blocks) of the experiment.

Rather than directly phenotype the RILs for toxicity, to avoid potentially mapping QTL for inbreeding depression, we chose to phenotype F1 individuals from matched crosses between pA1 males and pB2 females or pA2 males and pB1 females (Figure 1A). We arbitrarily crossed A/B RILs with the same line number (*i.e.*, pA1_1_*pB2_1_, … , pA1_n_*pB2_n_, pA2_1_*pB1_1_, … , pA2_m_*pB1_m_). We performed 396 pA1 male to pB2 female and 302 pA2 male to pB1 female crosses during the course of 8 weeks. For each cross, five virgin females from the pB population were mated to five males from the pA population in each of two vials with parents cleared after 4 d. The vials were left at 23° for 8 more days, and 12 F1 brother-sister mated adult males and females were collected for exposure to MTX. Generally all 24 flies exposed to MTX came from the same vial (~80% of the time), although occasionally we needed additional offspring from the backup vial. Each week we attempted ~120 crosses, although not all crosses produced enough offspring to proceed to the next step, leading to ~100 successful crosses per week.

Three females and three males were each placed in three “treatment cells” in a device we call a fly condo, and a fourth “control cell” was placed in a second condo. Fly condos consist of an array of “cells” with dimensions similar to a standard vial, except the top is a fine mesh screen, and the bottom (containing food) can be removed from the entire condo at one time. Experimentally the condos allow us to anesthetize an entire array of flies simultaneously and quickly change from standard food to MTX food. In the MTX experiment described here any given treatment condo contained four different crosses (= genotypes) each exposed in triplicate to MTX with each exposure cell having three males and three females exposed. Flies were placed in the condos and immediately exposed to MTX for 3 d. MTX (catalog no. M1676; LKT Laboratories, Inc,) was diluted in DMSO to 2.2 mM, then mixed with liquid food (12.5 g of sucrose, 17.5 g of active dry yeast, 5 mL of Karo light corn syrup, 95 mL phosphate-buffered saline; autoclaved before use) to a final concentration of 110 μΜ, and 100 μL was then spread onto a 1-cm × 2-cm piece of Whatman filter paper (cat. no. 3030 392) and placed into a 1-inch high agar plug at the bottom of each fly condo cell. We have previously shown this to be an effective drug delivery method (Kislukhin *et al.* 2012).

After the 3-d exposure, flies were anesthetized with CO_2_, the bottom of the condo containing the MTX was replaced with standard food, and flies were allowed to recover for 24 hr. After the 24-hr recovery, we provided the flies with new standard food, allowed them to lay eggs for 48 hr, and then discarded the adults. The progeny produced by the treated F1 females were given an additional 14 d to develop into adults, at which point we froze the condos, then removed the offspring by transferring them to a “sandwich” of two GBC SelfSeal Repositionable Letter Size Laminating Sheets, 3 mil. To ensure accuracy, we triple counted the offspring from each cell and used the mean over the three cells as our estimate of fecundity. Thus mated female fecundity after a 3-d exposure to MTX is our measure of toxicity of MTX. All of the phenotype data described here is available at http://FlyRILs.org and has been deposited in the Dryad Repository: http://dx.doi.org/10.5061/dryad.d20qc.

Control flies were handled the same as experimental flies except they were only exposed to a mock treatment consisting of dimethyl sulfoxide and liquid food. After the 3-d exposure, as opposed to a 24- hr recovery followed by 2 d to lay eggs, the mock treatment flies were given only 24 hr to lay eggs with no recovery. After 14 more days, we froze the condos containing the progeny of treated flies and visually inspected each chamber for the presence of flies. In most cases the cells were “saturated” (see Figure S1A). That is, they appeared to contain close to the maximum number of progeny a cell could support (generally >100 offspring), so they were not individually counted. We primarily used the control group to identify crosses that resulted in poor female fecundity—we removed crosses resulting in fewer than 50 offspring from further analysis.

We estimated the saturation point of the condo wells to determine the top-end of the “dynamic range” of our assay. To determine condo saturation, a total of 48 wells were filled with five pairs of flies each, and the flies were allowed to lay eggs for 2 d. After 2 wk, the number of offspring was counted to determine condo saturation (Figure S1A, light grey bars). We compare these saturated cells to the number of offspring we see in each cell in our experiment for the four blocks we analyzed. Two things are apparent from Figure S1A: (1) in the no drug saturation−treated cells (where females are likely to have laid >>100 viable eggs) we are seeing the vials beginning to saturate at between 100 and 125 offspring (that is ~10% of our saturated cells have between 100-125 offspring), and (2) despite the wells having a saturation point, the vast majority of MTX-treated cells show many fewer offspring. In our actual experiment we only have three females to attempt to have the majority of the vials well below saturation levels.

Overall fecundity knockdown at our optimal oral methotrexate dose (of 110 μΜ) was determined on the basis of the number of offspring laid by three pairs of F1 hybrid brother-sister−mated *D. melanogaster* from pA1xpB2 crosses carried out in half-pint round bottom glass stock bottles. We treated 16 genotypes (pA2_45_*pB1_45_, pA2_47_*pB1_47_, pA2_52_*pB1_52_, pA2_53_*pB1_53_, pA2_59_*pB1_59_, pA2_60_*pB1_60_, pA2_61_*pB1_61_, pA2_68_*pB1_68_, pA2_70_*pB1_70_, pA2_71_*pB1_71_, pA2_74_*pB1_74_, pA2_78_*pB1_78_, pA2_80_*pB1_80_, pA2_81_*pB1_81_, pA2_82_*pB1_82_, and pA2_83_*pB1_83_) using our standard dosing protocol in condos. Three treated sets of three male and three female flies per genotype were then allowed to lay eggs for 48 hr in condos. One mock-treated set of three male and three female flies per genotype was placed into a half-pint round-bottom glass stock bottle. The reason for allowing the mock treated flies to lay-out in bottles was to avoid saturation of the condos. After 2 wk, the offspring produced were counted to determine the control fecundity level (Figure S1B). The results from the bottles were compared with the numbers of offspring produced by the MTX treated pairs of the same genotypes in condos. At the dose used we appear to have a ~50% reduction in fecundity, with considerable bottle-to-bottle or genotype-to-genotype variation about that reduction.

The entire assay from start (crossing pA and pB RIL parents) to finish takes 6 wk; thus, the experiment was performed over eight overlapping weeks (Figure 1B). In the first 3 wk we attempted 396 pA1*pB2 crosses and in weeks four and five we attempted 302 pA2*pB1 crosses. Weeks six through eight were used to repeat crosses that failed the first time around.

### *A priori* candidate genes

**Figure 2:**
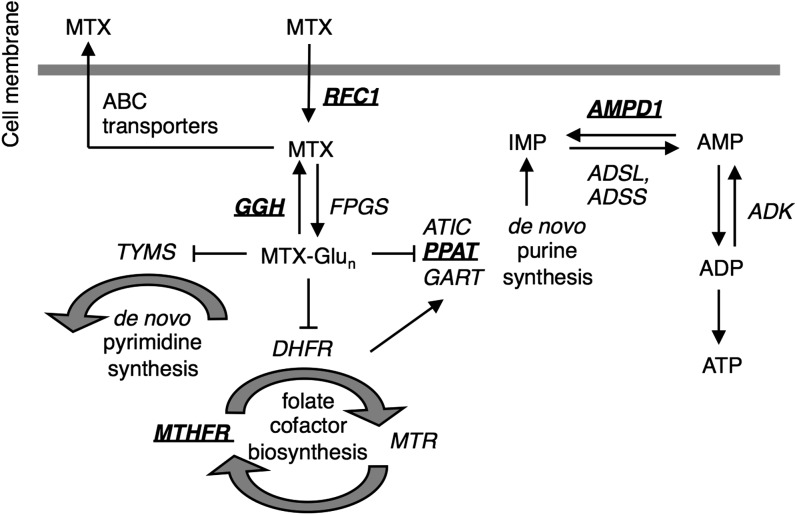
MTX cellular pathway. MTX enters cancer cells via the reduced folate carrier, and the efflux across the cell membrane is mediated by various ABC transporters. Inside the cell, MTX is converted to active methotrexate polyglutamates by folylpolyglutamate synthetase (FPGS), which adds glutamate residues to MTX. The primary action of MTX is inhibition of the enzyme dihydrofolate reductase (DHFR), which converts dihydrofolate to tetrahydrofolate. The effect of MTX depends on the function and expression of several other enzymes in the folate pathway, including methylenetetrahydrofolate dehydrogenase, 5,10-methylenetetrahydrofolate reductase, and thymidylate synthetase. Degradation of MTXPGs to MTX depends on the activity of the lysosomal enzyme GGH, which catalyzes the removal of polyglutamates (Mikkelsen *et al.* 2011). AMP deaminase (AMPD1) converts adenosine monophosphate (AMP) to inosine monophosphate (IMP). Adenylosuccinate synthase (ADSS) and adenylosuccinate lyase (ADSL) counteract the action of AMPD1, convert IMP back to AMP in a two-step process. Adenylate kinase catalyzes the formation of two molecules of ADP from AMP and ATP; ADP is the substrate for oxidative phosphorylation that forms ATP. GSTs are not known to directly interact with methotrexate but are main mediators of detoxification by conjugation of xenobiotics with glutathione.

Despite the fact chemotoxicity assays are difficult to perform in humans, it is possible to identify *a priori* human candidate genes for methotrexate toxicity based on the methotrexate cellular pathway (Figure 2, Table S2). We identified additional candidate genes from publications by using a PubMed search with key words “methotrexate”, “toxicity,” and “polymorphism.” The resulting set of 137 publications was then manually curated to identify only those publications in which the patient’s germ line genotype (not a tumor’s genotype) at a gene impacted toxicity (Table S2). We limited our search to studies in humans and did not include any studies in which the polymorphisms’ effects on toxicity were not statistically significant (there is considerable heterogeneity in the literature as to what warrants statistical significance). After identifying the *a priori* candidates, we used the ensembl.org browser to identify the orthologs of those genes in *D. melanogaster*; the ortholog types are listed along with *D. melanogaster* gene names in Table S2. The remaining human candidate genes listed in Table S2 are not associated with publications reporting a germline genetic polymorphism impacting MTX toxicity, although they are believed to be in the pathway that processes MTX, based on biochemical studies. The role of these “pathway implicated” genes is discussed most fully in a recent review by Mikkelsen *et al.* (2011). Glutathione *S*-transferase (GST) is a strong human *a priori* candidate gene family with known pharmogenetic polymorphisms, but Ensembl’s fly ortholog prediction seems incorrect, we define the human GST fly orthologs to be the two *Drosophila* regions harboring tandemly repeated GST gene families.

### Data analysis

All data analysis was performed in R (R Development Core Team 2012). We used a mixed-model analysis of variance by using the lme function in the nlme package (Pinheiro *et al.* 2012) to test for an effect of week (block) on MTX toxicity. Cross ID and week were random effects with cross ID nested within week. A likelihood ratio test showed a highly significant effect of week (χ_1_ = 397.9 *p* < 0.0001). Visually, weeks 6, 7, and 8 all had much lower knockdown of offspring, and the number of offspring produced was near condo saturation level (Figure 1C). Week 4 showed the opposite pattern, where the knockdown was too high with many of the females producing zero offspring (Figure 1C). Because we felt these weeks did not represent our target phenotype, in all subsequent analyses, we ignored these four weeks, and only used the weeks with mean fecundity knockdown near 50%, roughly the same trait studied in our earlier work (Kislukhin *et al.* 2012). Dropping weeks 4, 6, 7, and 8 reduced our total number of crosses/genotypes to 307 for all further analyses.

### Heritability and MTX QTLs

We estimated the broad sense heritability of MTX toxicity by estimating the genetic and phenotypic variance components from a linear mixed model using the lme and VarCorr functions in the nlme package (Pinheiro *et al.* 2012). Note that because all three replicates were reared in the same condo (although different cells), these heritability estimates include some microenvironmental effects. We estimated the heritability of cross means as the estimated genetic variance component over the total variance of cross means. Heritability estimates were obtained from the censored collection of 307 pA male to pB female crosses.

The general analytical framework for QTL mapping in the DSPR is described in King *et al.* (2012a,b). One of the features of the DSPR is that the underlying mosaic haplotype structure of every RIL is known with a high degree of certainty via an HMM (King *et al.* 2012b). For each position in each RIL in the DSPR panel, the HMM has assigned the probability that the founder genotype is 1 of the 36 possible founder genotype combinations (8 homozygous possibilities, 24 heterozygous possibilities). These assignments are made at evenly spaced positions across the genome, every 10 kb. The vast majority of the genomes of the RILs are homozygous, and we therefore converted the 36 founder genotype probabilities to eight additive probabilities by assuming any heterozygous states are intermediate to the two homozygous states. To perform QTL mapping, we regressed mean MTX toxicity for each cross on the 16 additive probabilities corresponding to the probabilities the paternal RIL was derived from each of the pA founders and the probabilities the maternal RIL was derived from each of the pB founders and included subpopulation as a covariate. We did not include the random effect of condo in the QTL analysis. Although any given condo held four specific crosses, founder genotypes at each position occur in many different crosses and thus across many different condos. In addition, given that there were only four crosses per condo and each cross occupied a single condo, cross ID and condo are confounded, and by including condo in the model as a covariate we likely remove some of the genetic variance we are attempting to explain. Thus, the model is as follows:

$$y=\mu +\beta_{s}S+\sum_{i=1}^{7}\beta_{A,i}G_{A,i}+\sum_{i=1}^{7}\beta_{B,i}G_{B,i},$$

where *µ* is the grand mean; *S* is subpopulation; *G_A,i_* are the genotype probabilities for the paternal RIL; *G_B,i_* are the genotype probabilities for the maternal RIL; and *β_s_*, *β_A,i_*, and *β_B,i_* are the corresponding effect estimates. Because we assayed females, the model for the X chromosome is the same as for the autosomes. Some founder genotypes were poorly represented in the crosses we assayed at some locations in the genome (*i.e.*, fewer than five crosses have that founder genotype at that genomic position). We found that including these terms, with little representation, in the model could lead to inflated *p*-values. Therefore, at a given position, if the sum of the probabilities across all the crosses assayed for a given founder genotype probability was less than 4.8, we dropped that term from the model (the result is that the model degrees of freedom vary with genomic position). Including week as a block effect gave very similar results (data not shown) and thus, for simplicity, we present the results from the model without the effect of week here. At each position, we calculated the *F*-statistic for the overall effect of genotype and obtained –log_10_(*p*-values). We then used the loess smoothing function in R with a span of 0.005 to smooth the –log_10_(*p*-values) across genetic distance to temper any highly localized fluctuations (*cf*. Paterson *et al.*, 1988).

We estimated the effects of each founder genotype at each QTL separately for each population (pA and pB). To do this, we fit the following model: $y_{r}=\sum_{i=1}^{8}\beta_{i}G_{i}$, where *y_r_* are the residuals after correcting for subpopulation, *G_i_* are the founder genotype probabilities, and *β_i_* are the corresponding effect estimates. Once again, only founder genotypes whose sum across all crosses was greater than 4.8 were included.

We performed 1000 permutations of the data to determine genomewide significance thresholds (Churchill and Doerge 1994) for several false-positive rates. For each permutation, we smoothed the resulting –log_10_(*p*-values) as we did for the observed data with the same loess smoothing function. We then used the peak finder function msPeakSimple from the msProcess library (Gong *et al.* 2012) in R with a span of 50 and a signal to noise threshold of 1 to identify distinct peaks across the genome. For a wide range of potential –log_10_(*p*-value) thresholds, we quantified the number of distinct peaks per genome scan for each permutation that exceeded that threshold. We could then calculate the number of observed peaks exceeding a given –log_10_(p-value) threshold per genome scan and determine a range of false positive rates. For example, the –log_10_(*p*-value) threshold that corresponds to 0.05 peaks per genome scan is our threshold corresponding to a 5% false positive rate. We show false-positive rates ranging from 0.05 to 3 expected peaks per genome scan.

We identified and localized peaks of interest using the smoothed –log_10_(*p*-values) as described previously. We considered a peak to be a peak of interest if one of the following conditions was met: (1) the peak colocalized with a *D. melanogaster* ortholog of a known MTX toxicity candidate gene identified *a priori* and had a *p*-value < 0.01 (see *Materials and Methods*), or (2) the –log_10_(p-value) exceeded our 0.5 genome-wide false-positive rate threshold (*i.e.*, a 50% chance of a single false-positive genome wide). To obtain confidence intervals on these peaks, we performed a genome scan without dropping founder genotype terms with poor representation and we converted the resulting *F*-statistic to a logarithm of odds (LOD) score (Broman and Sen 2009). We did not drop founder genotype terms when determining confidence intervals to keep the degrees of freedom for the model constant as variations in the degrees of freedom alter the relationship between LOD scores and *p*-values and alter the amount of LOD drop corresponding to a given percent confidence interval (Manichaikul *et al.* 2006; Broman and Sen 2009; King *et al.* 2012b). We used these LOD scores to calculate confidence intervals in two ways. First, we used a traditional 2 LOD drop interval. However, whereas 2 LOD intervals are conservative for two line crosses, the necessary LOD drop increases for larger degrees of freedom in the model (Manichaikul *et al.* 2006) and we have previously shown they are overly liberal for our 8-way crosses (King *et al.* 2012b). We also calculated Bayes credible intervals, for which 95% coverage is more consistent for different sample sizes, experimental designs, and effect sizes (Manichaikul *et al.* 2006; Broman and Sen 2009).

We explored multiple QTL models for subregions in which we appeared to identify two closely linked QTL. We performed both local 2D genome scans surrounding these regions and performed genome scans after statistically correcting for each of these QTL. In both cases we dropped founder genotype probabilities that did not sum to at least 4.8 as indicated previously. The local 2D scans were performed by fitting the following model:

$$y=\mu +\beta_{s}S+\sum_{i=1}^{7}\beta_{A,i,1}G_{A,i,1}+\sum_{i=1}^{7}\beta_{B,i,1}G_{B,i,1}+\sum_{i=1}^{7}\beta_{A,i,2}G_{A,i,2}+\sum_{i=1}^{7}\beta_{B,i,2}G_{B,i,2}$$

where *µ* is the grand mean; *S* is subpopulation; *G_i_* are the genotype probabilities for population A and B at position 1 and 2; and *β_s_*, *β_A,i,1_*, *β_B,i,1_*_,_ *β_A,i,2_*, and *β_B,i,2_* are the corresponding effect estimates. We considered a 14-cM region surrounding our QTL of interest and fit all possible pairs of positions in this region. Linkage limits our ability to distinguish very closely linked QTL and we only tested pairs of positions at least 2 cM apart. We also performed two genome scans after statistically correcting for two of our most significant QTL. We fit the following model:

$$y_{r}=\mu +\sum_{i=1}^{7}\beta_{A,i}G_{A,i}+\sum_{i=1}^{7}\beta_{B,i}G_{B,i},$$

where *y_r_* are the residuals from the best fitting single QTL model; *µ* is the grand mean; *G_i_* are the founder genotype probabilities for population A and B; and *β_s_*, *β_A,i_*, and *β_B,i_* are the corresponding effect estimates.

### Testing the effects of SNPs and transposable element (TE) inserts in candidate genes and candidate gene regions

The four QTL peaks identified were associated with seven candidate genes: A with *CG32626*, B with *GstE1–Gst E10* (a tandemly repeated gene complex), C with *PHGPx*, and D with *CG32154* and *CG32155*, *Gnf1*, *Prat*, and *pug* (the justification for these candidate genes is discussed more fully in the Results and Discussion). For each candidate gene we somewhat arbitrarily defined an upstream and downstream limit to that gene to be the region bounded by the next gene’s up- or down-stream coding region rounded to the nearest kilobase. These candidate gene regions are given in Table S3. Within each candidate gene region we identified three types of biallelic genetic polymorphisms: nonsynonymous SNPs (*i.e.*, SNPs predicted to encode an amino acid variant), other SNPs (including both synonymous SNPs, SNP in UTRs, and SNPs outside the coding regions), and segregating TE insertions. To determine whether amino acid−encoding SNPs explain linkage peaks, for QTL with one or more clear candidate genes, we identified all possible SNPs in the founder lines that are predicted to encode amino acid variants. For details on how we called SNPs in the founder lines, see King *et al.* (2012a; http://FlyRILs.org). We then inferred the probability each RIL harbored the minor allele and assigned a genotype value to each cross by adding the paternal and maternal probabilities. In the case of perfect certainty, genotype values are: 2 = AA, 1 = Aa, and 0 = aa. We then fit the following single marker model for each possible amino acid variant:

$$y=\mu +\beta_{s}S+\beta_{m}M,$$

where *S* is subpopulation, *M* is the cross genotype at the marker, and *β_s_* and *β_m_* are the corresponding effect estimates. We also fit this model for 50 randomly selected SNPs that fell within the widest confidence interval for each identified QTL to obtain a null expectation for SNPs linked with our QTL. We carried out a similar analysis for the nonsynonymous SNPs in the candidate gene intervals. We additionally had access to a set of unpublished annotations of TE insertions segregating in the founders (J. M. Cridland, S. J. MacDonald, A. D. Long, and K. R. Thornton, unpublished results), of which there were four segregating in candidate gene intervals (at X:13727665, 2R:14293010, 2R:14294252, and 3L:16227431 in A, B, B, and D, respectively), we also tested each of these TE insertions for an association with MTX toxicity. Aside from correcting for subpopulation, we did not correct for kinship among the lines. Within subpopulations, kinship is fairly even among the lines and we do not expect it to have a large effect.

## Results

**Figure 3:**
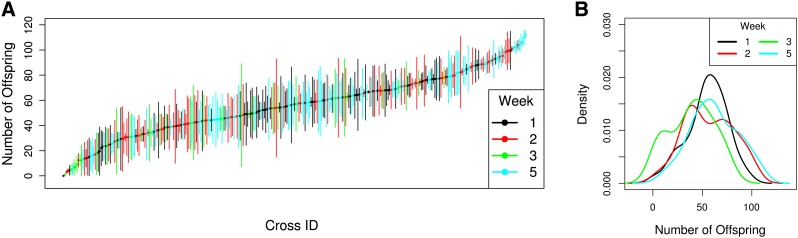
(A) Means and SEs of the number of offspring produced by each F1 female from each pA-pB cross. Different colors correspond to different weeks (blocks) of the experiment after removing weeks with poor dosing. (B) Density plot of the mean number of offspring produced by the three replicate females from each RIL. Different colors correspond to different weeks (blocks) of the experiment.

We used female fecundity as a measure of MTX chemotoxicity in 698 pA RIL male by pB female crosses over eight experimental blocks (Figure 1B). The fecundity of a pool of three females in three replicate vials was assayed for each genotype (Figure 1A). Female fecundity is a reliable indicator of heritable toxicity (Kislukhin *et al.* 2012). For reasons beyond our experimental control three of the weeks (blocks) showed very little fecundity knockdown (with counts approaching vial saturation levels), and a fourth week (block) showed extremely high knockdown (Figure 1C). Although we cannot pinpoint the exact cause of this shift in mean fecundity over time, we propose that the drug−food solution was not homogeneous in those weeks, where some condo wells had almost no drug, whereas the other wells had much greater concentrations. We decided to remove these blocks from further consideration to ensure we were mapping QTL associated with a knockdown of ~50%, this decision results in a dataset consisting of 307 RIL cross pairs (Figure 3). Substantial variation existed among crosses in MTX toxicity as measured by the number of offspring produced after MTX exposure (Figure 3). We estimated broad sense heritability of MTX toxicity to be 0.38. For QTL mapping, we used the mean MTX toxicity (over the three replicate vials per cross) and thus we also estimated the broad sense heritability of genotype mean MTX toxicity, which is 0.64. Both of these estimates are much lower than the narrow sense heritability we estimated in a previous study using a similar assay (Kislukhin *et al.* 2012; see *Discussion*).

**Figure 4:**
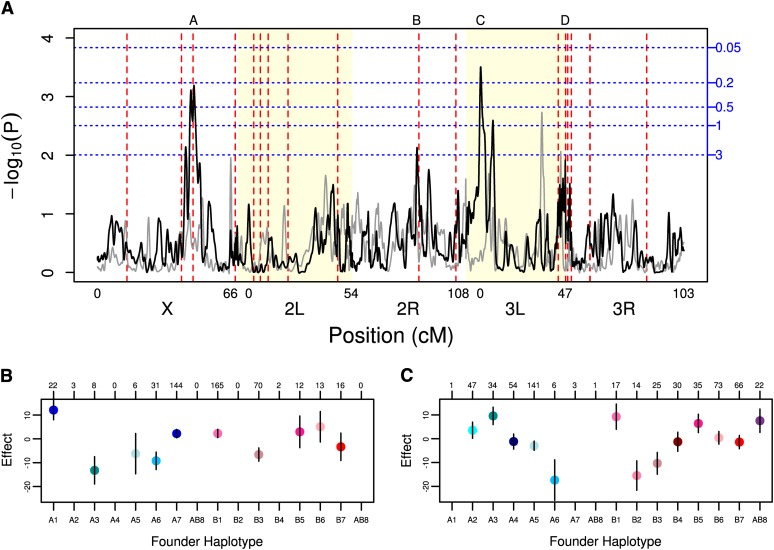
(A) MTX toxicity genome scan. The major chromosome arms are delineated by different background shading (white/yellow). The black line is the scan of the observed data. To give an example of the results obtainable by chance alone, the grey line is a scan of a single permutation of the observed data. Horizontal blue dotted lines indicate thresholds for various false positive rates (number of expected peaks per genome scan) obtained via permutations. Vertical dashed red lines indicate the location of fly orthologs of previously identified candidate genes for methotrexate toxicity. (B-C) Standardized founder haplotype means and SE at (B) QTL A and (C) QTL C. The number of each confidently assigned (probability > 0.95) founder genotype is listed above each plot. Only means with at least five observations are plotted.

Our genome scan identified three QTL regions of interest, labeled A-C (Table 1; Figure 4A). Two of these QTL (A and C) exceed the 0.5 genome-wide false-positive threshold (given that the expectation at this threshold is 0.5 false positives and we observe 2 QTL, this corresponds to a false discovery rate of 25%) with QTL C, the most significant peak, also exceeding the 0.2 threshold. QTL B did not exceed the 0.5 threshold; however, it corresponds to known candidate gene (see below), and had a marginal *p*-value of 0.007 so it of interest. An additional QTL region, QTL region D, was not significant as determined by our criteria, and it spans the centromere of chromosome *3* (so resolution is poor); nonetheless we discuss it in this section as the interval defined by this QTL includes several *a priori* candidate genes and the architecture of the peak mirrors this cluster of candidate genes. These three QTL each explain between 15% and 20% of the genetic variation in MTX toxicity (Table 1), and together they explain ~45% of the total genetic variation. The total genetic variation explained is very similar if we fit a model that fits all three QTL simultaneously, or simply sum over the variance explained by each QTL individually, not an unexpected result given the large number of genotypes and unlinked QTL. We were able to resolve QTL A, B, and C to ~3 – 4 cM (~700−900 kb, Table 1). QTL D is a wide peak that spans the centromere of chromosome *3* and therefore resolution for this QTL was much lower at ~5–8 cM (~12–17 Mb; Table 1).

**Table 1:** Details of identified QTL

Name	Peak Location, Mb		-log_10_(*p*-Value)	2-LOD CI, Mb*^a^*	2-LOD CI, cM*^a^*	BCI, Mb*^b^*	BCI, cM*^b^*	Percent of H^2^*^c^*
A	*X*	13.85	3.19	13.25–14.60	43.01–48.37	13.48–14.31	44.02–47.35	0.15
B	*2R*	14.00	2.13	13.82–14.38	84.06–85.72	13.84–14.31	84.12–85.52	0.12
C	*3L*	3.42	3.5	2.79–3.84	4.61–8.58	3.02–3.98	5.45–9.11	0.19
D	*3L*	22.24	1.92	*3L:*17.76–*3R*:5.85	44.76–49.25	*3L:*14.78–*3R*:7.5	42.08–49.25	0.10

QTL, quantitative trait loci.

a 2-LOD CI indicates 2 LOD support intervals.

b BCI are Bayesian credible intervals.

c Percent of H2 refers to the percent of broad sense heritability of cross means.

We estimated the phenotype effects of each founder haplotype on MTX toxicity for the two QTL exceeding our 0.5 genome-wide false-positive rate. The estimated effects of each founder genotype do not show a pattern suggesting simple biallelism (Figure 4, B and C). However, factors such as multiple linked QTL in the region, multiple alleles for a single causative gene, non-additivity of founder genotypes, or variation in the frequency of founder genotypes in the two populations could make the effects at our QTL difficult to estimate or interpret.

The A and C QTL regions both showed smaller nearby peaks (Figure 4A). In both cases the satellite peak was close to the major peak and separated by a trough in likelihood. To help determine whether these loci represented multiple distinct QTL, we performed local 2D genome scans and full genome scans after statistically controlling for each of QTL A and C. Linkage limits our ability to distinguish very closely linked QTL and we only tested pairs of positions at least 2 cM apart. The local 2D scans confirmed the single QTL results, showing the most significant results only when the most significant single QTL was included in the model. The most significant *p*-value for both local 2D scans (QTL A: −log_10_(P) = 4.39; QTL C: −log_10_(P) = 4.44) was not substantially more significant than our most significant single QTL models (QTL A: -log_10_(P) = 3.19; QTL C: −log_10_(P) = 3.5). In addition, performing genome scans after statistically correcting for each of QTL A and C (Figure S2) did not show any additional peaks in the region of the QTL corrected for, nor did we observe any new QTL co-localizing with known candidate genes or exceeding our 0.5 threshold. Given we do not have strong evidence that QTL regions A and C represent multiple QTL, we treat them as single regions, however, it is possible future studies with larger sample sizes may reveal multiple QTL in these regions.

### Two of the three mapped, QTL are associated with fly orthologs of human genes believed to be important in MTX toxicity:

We identified a total of three significant (at our criteria outlined previously) QTL, labeled A-C (Figure 4A; Table 1). Of these three, two lie directly over the fly orthologs of human genes in the methotrexate pathway (Table S2; Table S3; Figure S3). These three QTL encompass 2.46MB in total (the sum of the Bayesian credible intervals of Table 1). Including only regions of the genome with typical levels of recombination (X: 2.5−21 Mb, 2L: 1−17.5 Mb, 2R: 7−19 Mb, 3L: 1−19 Mb, 3R: 7−24 Mb), where it is possible to genetically locate to submegabase scales, the fine-map-capable fly genome is 82 Mb. Because we have 15 fly orthologs of human candidate genes in the 82-Mb fine-map-capable fly genome (Table S2), the probability of observing two or more candidate genes under of mapped peaks is 7.5% (*i.e.*, 1 − ppois(1,15*(2.46/82))). Although this is not a formally significant enrichment for candidate genes, a *p*-value of 7.5% is suggestive of enrichment. In addition, the particular candidate genes are very strong because they harbor pharmacologically relevant genetic polymorphisms that impact MTX toxicity and are in the MTX toxicity pathway (Table S2). If we ask whether there is an enrichment for the subset of six candidate genes that have pharmacologically relevant polymorphisms and fly orthologs the p-value for enrichment is 1.4% (*i.e.*, 1 − ppois(1,15*(2.46/82))).

QTL A includes the candidate gene *CG32626*, an ortholog of the human gene *adenosine monophosphate deaminase 1* (*AMPD1*). Patients with rheumatoid arthritis treated with methotrexate carrying the C34T allele of the *AMPD1* gene were shown to have better clinical response to the drug (Wessels *et al.* 2006). QTL B lies over a gene family, *GSTE1−GSTE10*, consisting of orthologs of the *glutathione S transferase* (*GST*) gene, previously associated with high hepatotoxicity in lymphoblastic leukemia and malignant lymphoma methotrexate treatment (Imanishi *et al.* 2007).

The rather broadly peaked QTL D is not significant based on our criteria and spans a centromere but nonetheless includes several candidate genes. The peak is much broader than those typically observed so it is conceivable this peak is associated with multiple linked genes. Genes *CG32154* and *CG32155* are orthologs of the human *Gamma-glutamyl hydrolase* (*GGH*) gene. This gene is involved in the methotrexate pathway (Figure 2) and has been shown to be associated with methotrexate efficacy and toxicity in Japanese patients with articular-type juvenile idiopathic arthritis (Yanagimachi *et al.* 2011). Gene *Gnf1* is a 1-to-1 ortholog of the *human replication factor C subunit 1* (*RFC-1*) gene, which encodes a major methotrexate transporter. The mutation G80A in *RFC-1* has been associated with post-methotrexate treatment survival of children with acute lymphoblastic leukemia. Children with the GG genotype had better prognoses than those with the GA, and the AA genotype had the worst outcome (Laverdiere *et al.* 2002). *Prat* is an ortholog of the human gene *PPAT*, which is a member of the purine/pyrimidine phosphoribosyltransferase family. *PPAT* along with *GART* and *ATIC* is involved in the *de novo* purine synthesis of the methotrexate pathway (Figure 2). The *D. melanogaster* gene *pug* is an ortholog of the human *methylenetetrahydrofolate dehydrogenase 1* (*MTHFD1*) gene, which is part of the methotrexate pathway (Figure 2). Additionally, the C677T mutation in this gene has been associated with hematologic and liver toxicity during consolidation and maintenance treatment toxicity in acute lymphoblastic leukemia in children (Costea *et al.* 2006).

### QTL C identifies novel candidate genes for MTX toxicity

QTL C spans 122 fly genes, none are fly orthologs of our human *a priori* candidate genes (Table S2). In the absence of *a priori* candidates for this peak, genes worthy of additional consideration were determined by manual curation of the region. We were able to identify a single gene that appears to be closely related to methotrexate activity: fly gene *PHGPx*, having human ortholog *GPx4* (Imai and Nakagawa 2002). *GPx4*, which codes for the glutathione peroxidase protein, is a unique antioxidant enzyme shown to reduce phospholipid hydroperoxide in mammalian cells (Imai and Nakagawa 2002). This gene has decreased expression levels in renal rat tissue (Oktem *et al.* 2006) and in the spinal cords of rabbits (Ayromlou 2011) in the presence of methotrexate.

### QTL peaks are not due to amino acid polymorphisms in candidate genes

Predicted amino acid encoding variants in our seven candidate genes/gene regions (Table S3) explain only a modest amount of the heritability of cross means (ranging from near 0% to 3.8%) and much less than the QTL themselves, explaining at most 25% of the variance explained by any given QTL. We also tested a random set of 50 SNPs that fell within the widest confidence interval of each peak. In all cases, at least one of the randomly selected SNPs explained a larger proportion of the heritability than all of our identified potential amino acid variants. We therefore conclude our QTL are unlikely to be explained by coding variants in the candidate genes we identified.

### Noncoding SNPs in candidate gene regions may be causative

For all SNPs segregating among the founders we inferred genotypes in the RILs and carried out a gene-centric association study. By focusing on small genomic intervals containing only a handful of SNPs, we found that the statistical threshold for significance is greatly reduced. These gene-centric association scans are depicted in Figure S4, and the subset of SNPs having suggestive significance are presented in Table S4. One SNP in *CG32626* gene region (QTL A; base 13739344) reached Bonferroni significance and may explain as much as 10% of the genetic variation in MTX toxicity. Upon closer inspection of the raw Illumina read data this “SNP” is actually a small insertion deletion polymorphism with 8 of the 15 founders having deletions of a “G” rich tract ranging from 26 to 38 bases in size (Figure S5). This INDEL is ~50 bp downstream of the donor site for the five prime most first exon of *CG32626*. Because this gene is annotated using four different first exons, we hypothesize that it may impact splicing in some manner. A second SNP in the *PHGPx* gene region (3L:3339692) did not reach Bonferroni significance but was highly suggestive. This SNP is located downstream of this gene (which is located entirely in the intron of another gene) in a region that modENCODE annotates as an “active promotor/transcription start site” in both S2 and BG3 cells.

### Insertions of TEs in candidate gene regions

We tested for associations between four TE located in the candidate gene regions and MTX toxicity. Neither TE under QTL B was significantly associated with MTX toxicity, although both the TE under QTL A (X:13727665) and QTL D1 (3L:16227431) were significantly associated at p<0.0004 and p<0.0027 respectively. But these p-values must be interpreted with caution, in the DSPR it is difficult to attach meaning to an event only present in a single founder, since that event tags an entire local haplotype. That being said, the TE associated with QTL A is located in ~2 kb intron shared by all four alternatively transcripts of *CG32626*, so it may very well have a phenotypic effect. Similarly the TE under QTL D1 is located in the 650 bp ′UTR of *CG32155*, so is also likely to have a phenotypic effect. Interestingly, though the TE association with QTL A is plausibly implicated as being causative, it neither explains to total variation explained by the QTL, nor the variation among founder means at the QTL (*e.g.*, Figure 4B). This finding suggests that this QTL is multiallelic, with some of the variation explained by a rare TE of large effect and the remaining variation explained by regulatory SNP(s).

## Discussion

Using the DSPR panel, we were able to identify three QTL, two of which lie directly over the *D. melanogaster* orthologs of known human MTX candidate genes (QTL A and B; Figure 4A). This study validates the use of *Drosophila* as a model system for chemotoxicity and is perhaps the first example of standing phenotypic variation in flies and humans being due to standing variation in the same genes Furthermore, we identify a fly gene (with a human ortholog) that represents a new candidate gene worthy of additional study: *PHGPx* (human ortholog *GPx*) (QTL C; Figure 4A). All together, our three identified QTL explain 45% of the genetic variance in MTX toxicity. However, given the known upward bias in effect sizes for detected QTL (*i.e.* the Beavis effect), which is roughly proportional to the number of quasi-independent statistical tests divided by the number of inbred lines under study (Xu 2003), this estimate should be taken as an upper limit. Overall, we have verified that *Drosophila* can serve as a model to identify novel candidate genes important in chemotoxicity.

### Chemotoxicity in flies may be due to regulatory changes in fly orthologs of human candidate genes

Thus far, all of the candidate polymorphisms in humans that have been studied represent amino acid variants (see Table S2). However, the nonsynonymous SNPs in fly orthologs of human candidate genes under peaks fail to explain the observed mapped segregating variation in MTX toxicity. This finding suggests regulatory variants, controlling the expression of these genes, are more likely the mapped QTL. Although we are unable to definitively identify the causative variants for our identified QTL, this result does suggest current human pharmacogenomics efforts that focus on amino acid encoding variants are only scratching the surface of the genetic basis of chemotoxicity. One of the advantages to using a stable panel of RILs designed to be a community resource is the ability to assay multiple traits on the same panel and integrate data across these studies. In this case, gene expression data, especially utilizing ovary tissue, would be invaluable in distinguishing between coding and regulatory changes as the causative variation. As these types of data become available, future studies can integrate expression QTL data to help answer this question.

### Is the genetic basis of chemotoxicity due to an allelic series at each candidate gene?

When we test the complete set of biallelic SNPs in putative candidate gene regions under a QTL peak, it is difficult to identify a single SNP that explains the complete linkage signal. In the case of QTL D perhaps this is not surprising, the broad peak in combination with several excellent candidate genes clustering in this region suggests the mapped QTL could be due to the underlying effects of polymorphisms at several genes. That said, in the case of the other three mapped genes, a single gene explaining the peak seems far more parsimonious. The observation that no single SNP explains a given linkage signal, and the additional observation that it appears that our estimated haplotype effects do not nicely fall into two bins, suggests that more than two variants are segregating in the DSRP at each candidate gene. This is consistent with our first proof-of-principal publication from the DSRP (King *et al.* 2012a) where the statistical analysis suggested more than two alleles segregating at ADH explain ADH activity levels. It remains to be seen how often mapped QTL support this heterogeneity or allelic series model for the genetic architecture of complex traits.

Unreliability in the MTX dosing over blocks of the experiment limited our sample size (see methods) and therefore our power to detect QTL. The unreliability in dosing required us to discard several experimental blocks in which the flies were obviously being consistently over- or under-dosed, and likely resulted in greater heterogeneity in dosing within blocks than that seen in our earlier work (Kislukhin *et al.* 2012). This heterogeneity resulted in lower broad-sense heritabilities in this study than narrow-sense heritabilities observed in Kislukhin *et al.* (2012). Genome-wide studies that include large numbers of statistical tests are subject to stringent corrections for multiple tests to minimize type I error. At the same time, the high threshold for statistical significance results in high type II error. Here, we have attempted to present a more nuanced interpretation of our results considering both type I and type II error rates. For example, we do not subject peaks that fall directly over candidate genes to the same high threshold given the *a priori* expectation of a QTL at those locations. Our experimental design, crossing pA and pB RILs and measuring F1 individuals, requires us to fit a model with more degrees of freedom then other designs that could be considered, which also negatively impacts our power (King *et al.* 2012b). However, we believe some sort of outcrossing design is critical when measuring a life history trait such as post-treatment fecundity. Any direct measurements on the RILs carry the risk of mapping variation for inbreeding depression and this risk is especially high for life history traits.

Although we identified only three QTL using our model and criteria for significance, we likely missed many more genes known to be involved in the methotrexate pathway and methotrexate toxicity. Simulation studies show that we need to assay upward of 800 line crosses to reliably detect QTL contributing 5% to segregating variation (*c.f*. King *et al.* 2011b). To improve the power of our study in the future, the number of crosses used should be maximized and we should future optimize drug delivery. We feel with improvement in our first generation assays of toxicity, such gains can be realistically achieved. Our assay may also miss some genes completely if our original 15 founders are not segregating functional variants of measurable effect. Along this line a recent publication (Zichner *et al.* 2013) showed that the DGRP (Mackay *et al.* 2012) is segregating a presence/absence polymorphism of *GstE5*, whereas we see no evidence for this event in the DSPR. If this presence/absence polymorphism impacted MTX toxicity it could not be mapped in the DSPR.

It is clear from this study that *Drosophila* is a powerful model system for identifying genetic variants underlying traits of human interest. In addition to showing a similar genetic basis for MTX toxicity between humans and *Drosophila*, we were able to identify one novel MTX toxicity human target gene, *GPx*. Given it is currently unclear how this gene fits into the pharmacodynamic and/or the pharmacokinetic pathway of the drug (Figure 2), an exciting avenue for future research is confirming the role of this gene in human MTX toxicity and characterizing its role in the pathway.

## Supplementary Material

Supporting Information
