# Supplementary material for: The Genetic Architecture of Methotrexate Toxicity Is Similar in Drosophila melanogaster and Humans
Source: G3 (Bethesda). 2013 Aug 1;3(8):1301–10. doi: 10.1534/g3.113.006619 (PMC3737169; doi:10.1534/g3.113.006619)
Supplement: Supporting Information [file supp_3_8_1301__index.html]

The Genetic Architecture of Methotrexate Toxicity Is Similar in Drosophila melanogaster and Humans — Supporting Information 

# The Genetic Architecture of Methotrexate Toxicity Is Similar in *Drosophila melanogaster* and Humans

## Supporting Information for Kislukhin *et al.*, 2013

**Files in this Data Supplement:**

- Supporting Information - Figures S1-S5, Tables S1-S4, and Supporting References (PDF, 3 MB)
- Figure S1 - Saturation and total percent knockdown (PDF, 131 KB)
- Supporting References - PDF, 175 KB
- Figure S2 - Residual methotrexate toxicity genome scans adjusting for a) QTL A and b) QTL C (PDF, 238 KB)
- Figure S3 - Santa Cruz Genome Browser for Bayesian Credible Intervals (Table1) for QTLs A-C (PDF, 1.3 MB)
- Figure S4 - Association scans with MTX toxicity for all SNPs in candidate genes listed in Table S3 (PDF, 401 KB)
- Figure S5 - a) Alignment of INDEL polymorphism immediately downstream of exon 1 of CG32626 (the candidate gene for QTL A). b) Santa Cruz Genome Browser screen shot of CG32626 showing four putative alternate first exons. (PDF, 617 KB)
- Table S1 - Recovery and "lay-out" Fly Food (PDF, 60 KB)
- Table S2 - Methotrexate *a priori* identified polymorphisms (PDF, 80 KB)
- Table S3 - Candidate genes associated with QTL peaks of Figure 3A and Table 1. (PDF, 67 KB)
- Table S4 - Biallelic SNPs significant a p<0.001 from gene-centric association scans (PDF, 71 KB)
