# Supplementary material for: The Genetic Architecture of Methotrexate Toxicity Is Similar in Drosophila melanogaster and Humans
Source: G3 (Bethesda). 2013 Aug 1;3(8):1301–10. doi: 10.1534/g3.113.006619 (PMC3737169; doi:10.1534/g3.113.006619)
Supplement: Supporting Information [file supp_g3.113.006619_006619SI.pdf]

G3 · Genes | Genomes | Genetics

The genetic architecture of methotrexate toxicity is similar in *Drosophila melanogaster* and humans.

Authors: Galina Kislukhin*[1], Elizabeth G. King*[1,3], Kelli N. Walters[1], Stuart J. Macdonald[2], Anthony D. Long[1]
*Co-first Authors

[1]University of California, Irvine, Department of Ecology and Evolutionary Biology, Irvine, CA 92697-2525, and [2]University of Kansas, Department of Molecular Biosciences, Lawrence, KS 66045
[3]Corresponding Author: University of California, Irvine, Department of Ecology and Evolutionary Biology, Irvine, CA 92697-2525. E-mail: egking@uci.edu

DOI: 10.1534/g3.113.006619

a

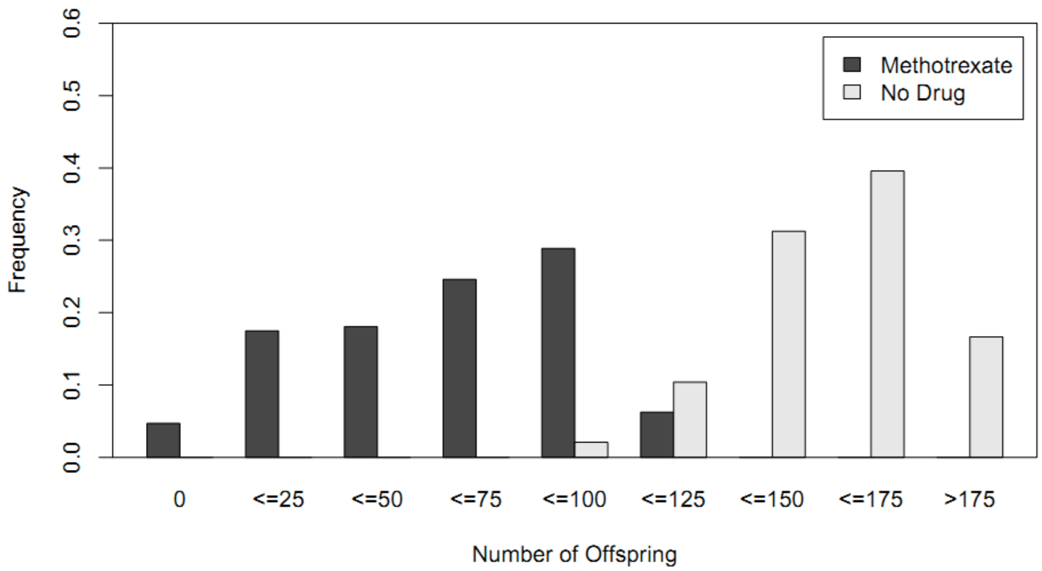

b

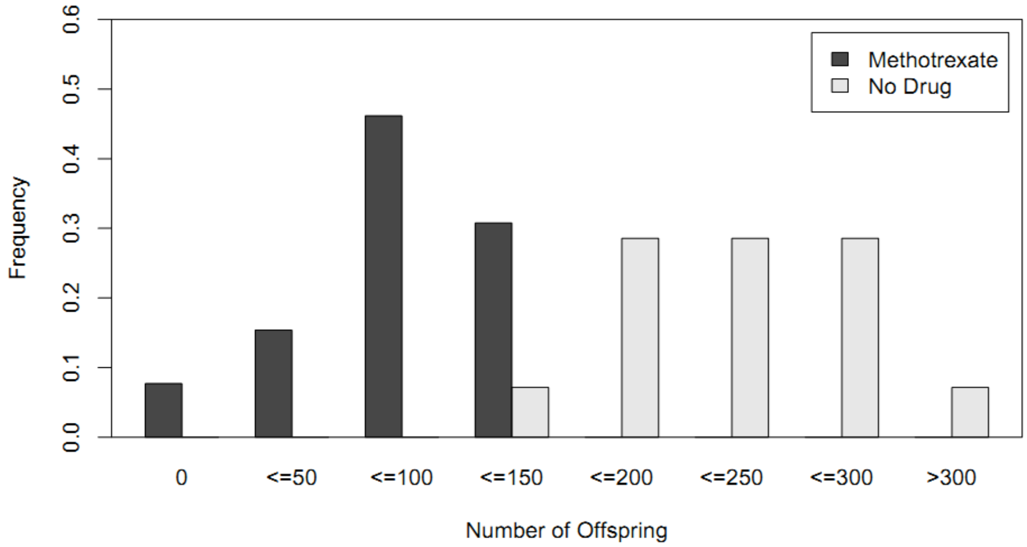

Figure S1 Saturation and total percent knockdown. **a)** Saturation: A "no drug" treatment was carried out in fly condos in order to assess the saturation point of the condo wells. A total of 48 wells were filled with 5 pairs of flies each, and allowed to lay eggs for 2 days. In two weeks, the number of offspring was counted and compared to the number of offspring produced by all RIL crosses of the MTX-treated assay. **b)** Percent knock-down: 16 genotypes were treated using our standard dosing protocol in condos. Three treated sets of 3 males and 3 females per genotype were then allowed to lay eggs for 48 hours in condos. One mock treated set of 3 males and 3 females per genotype were also allowed to lay eggs in bottles for 48 hours. We compare the mean of each treated genotype (dark grey) to the same genotype from the mock-treated fecundity assayed in half-pint round-bottom glass stock bottles (light grey).

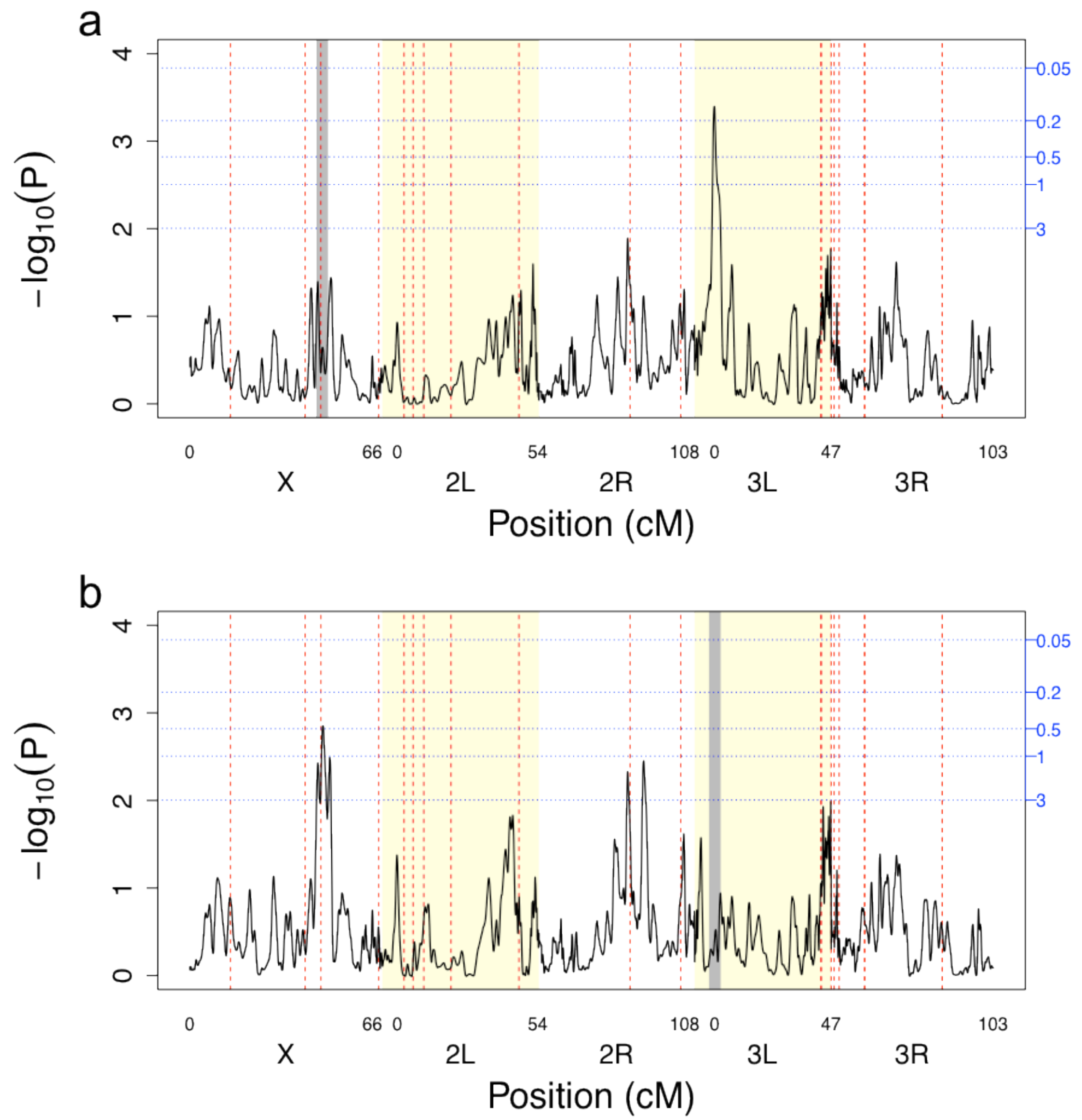

Figure S2 Residual methotrexate toxicity genome scans adjusting for **a)** QTL A and **b)** QTL C. The grey vertical bar indicates the region within 2cM of the QTL corrected for and therefore where results should be interpreted with caution. Horizontal blue dotted lines indicate thresholds for various false positive rates (number of expected peaks per genome scan) noted on the right y-axis. Vertical dashed red lines indicate the location of fly orthologs of previously identified human candidate genes for methotrexate toxicity.

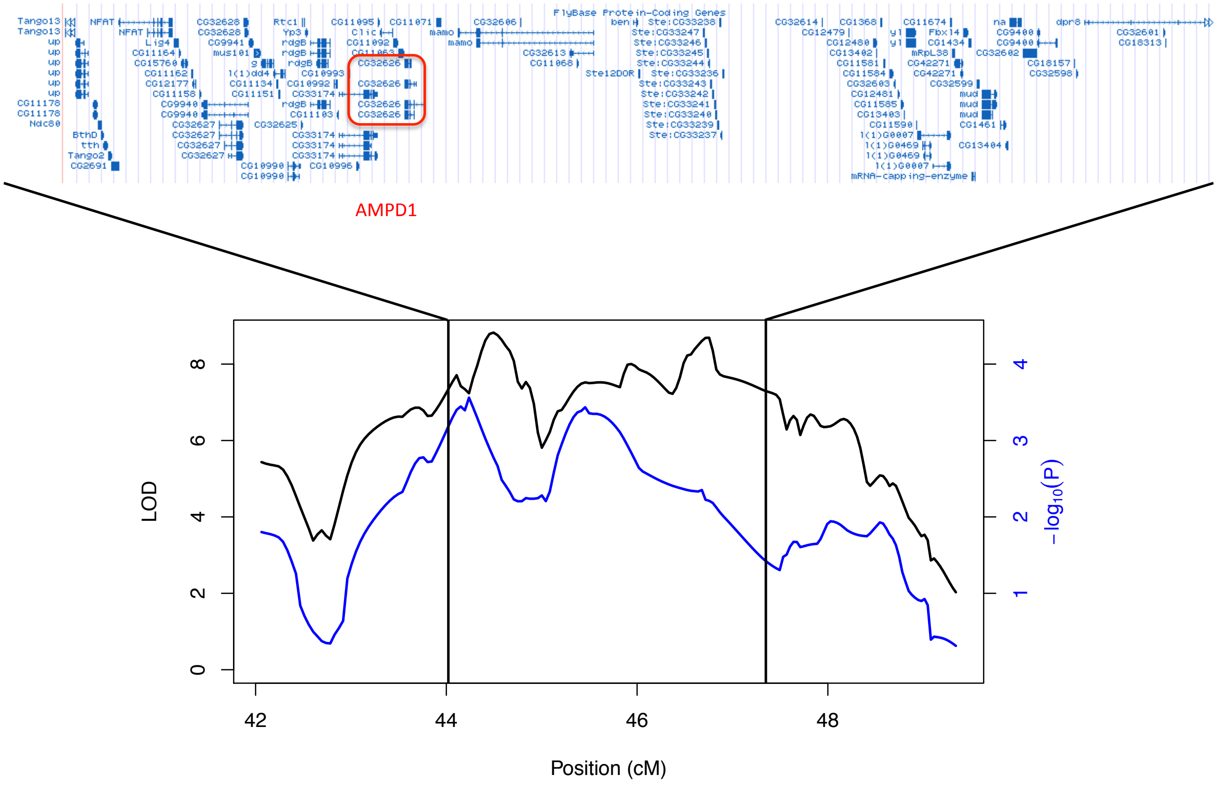
FlyBase Protein-Coding Genes
AMPD1
LOD
−log10(P)
Position (cM)
42
44
46
48
0
2
4
6
8
1
2
3
4

QTL A

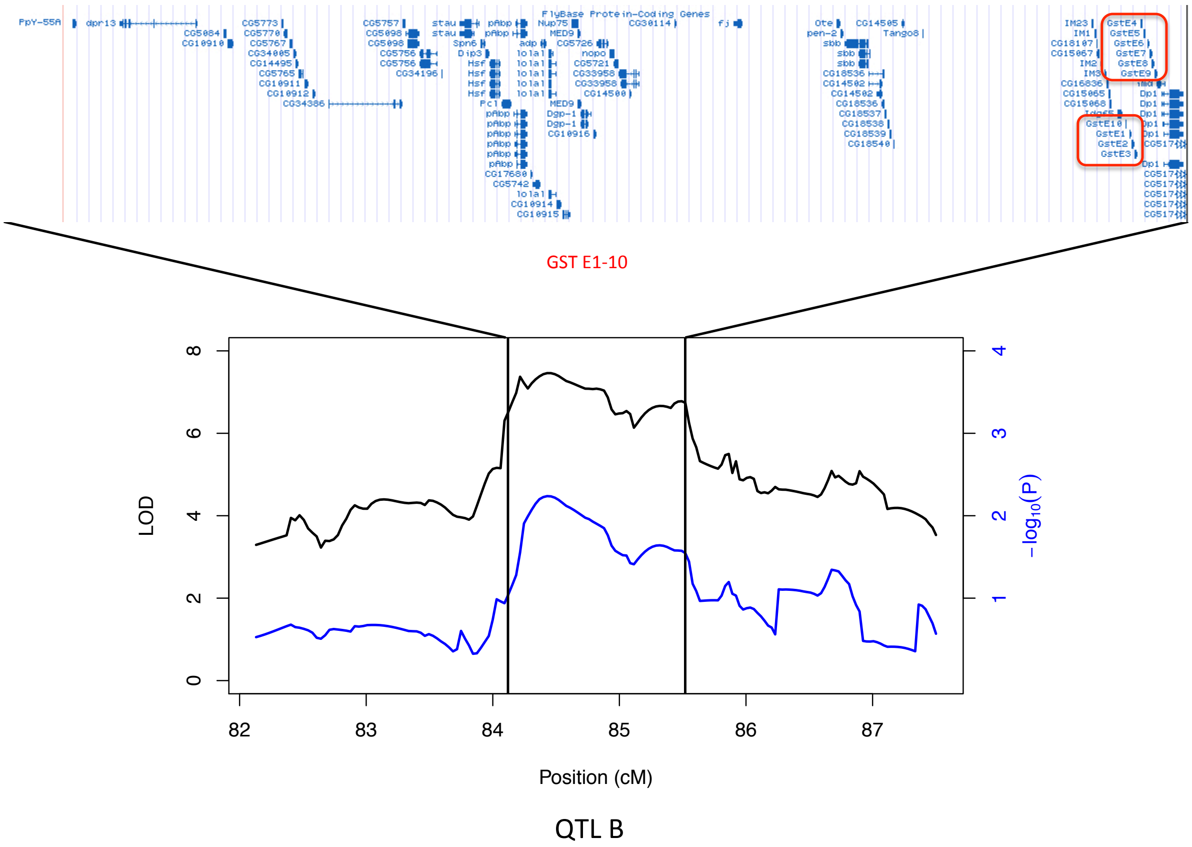
FlyBase Protein-Coding Genes
GST E1-10
LOD
−log10(P)
Position (cM)
QTL B

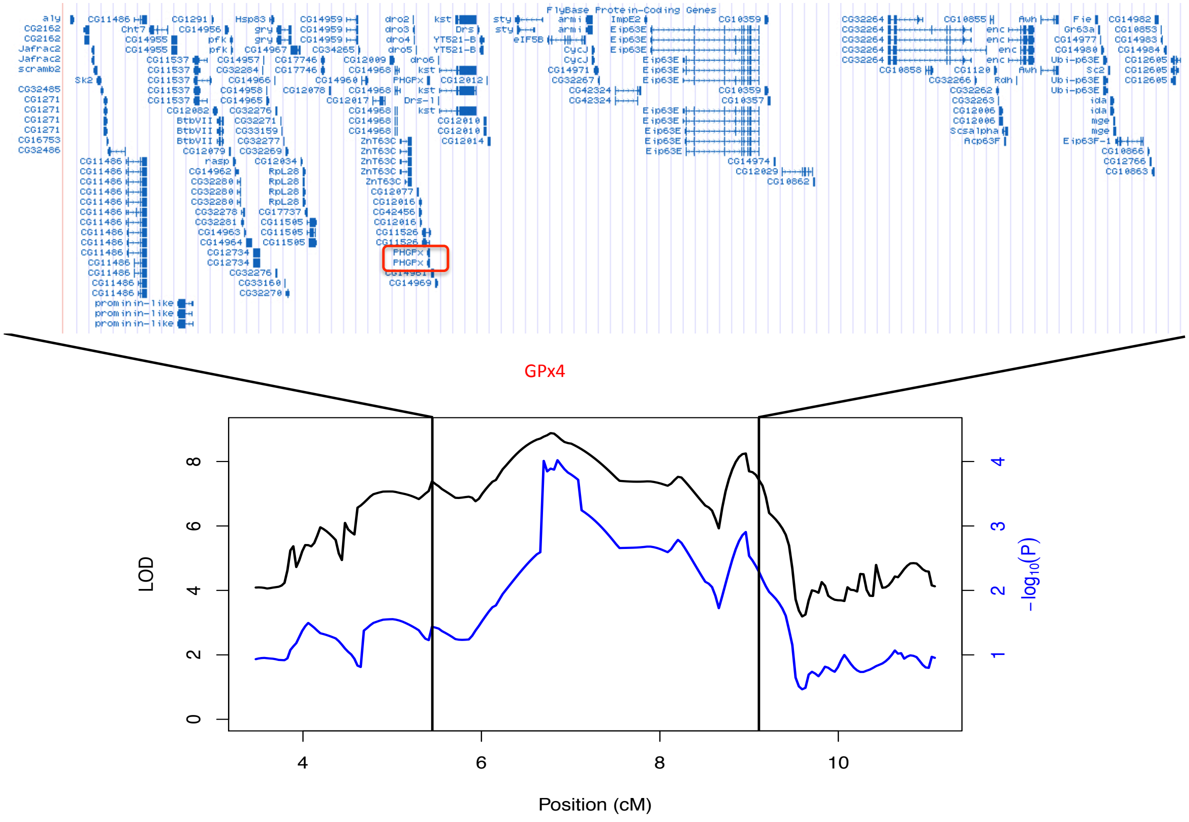

QTL C

Figure S3 Santa Cruz Genome Browser for Bayesian Credible Intervals (Table1) for QTLs A-C. QTL D spans a centromere so is poorly resolved. For QTLs A&B the Drosophila orthologs of a priori human candidate genes is circled in red with the associated human gene name also in red. For QTL C the novel candidate gene is circled in red, with the associated human genes name also given.

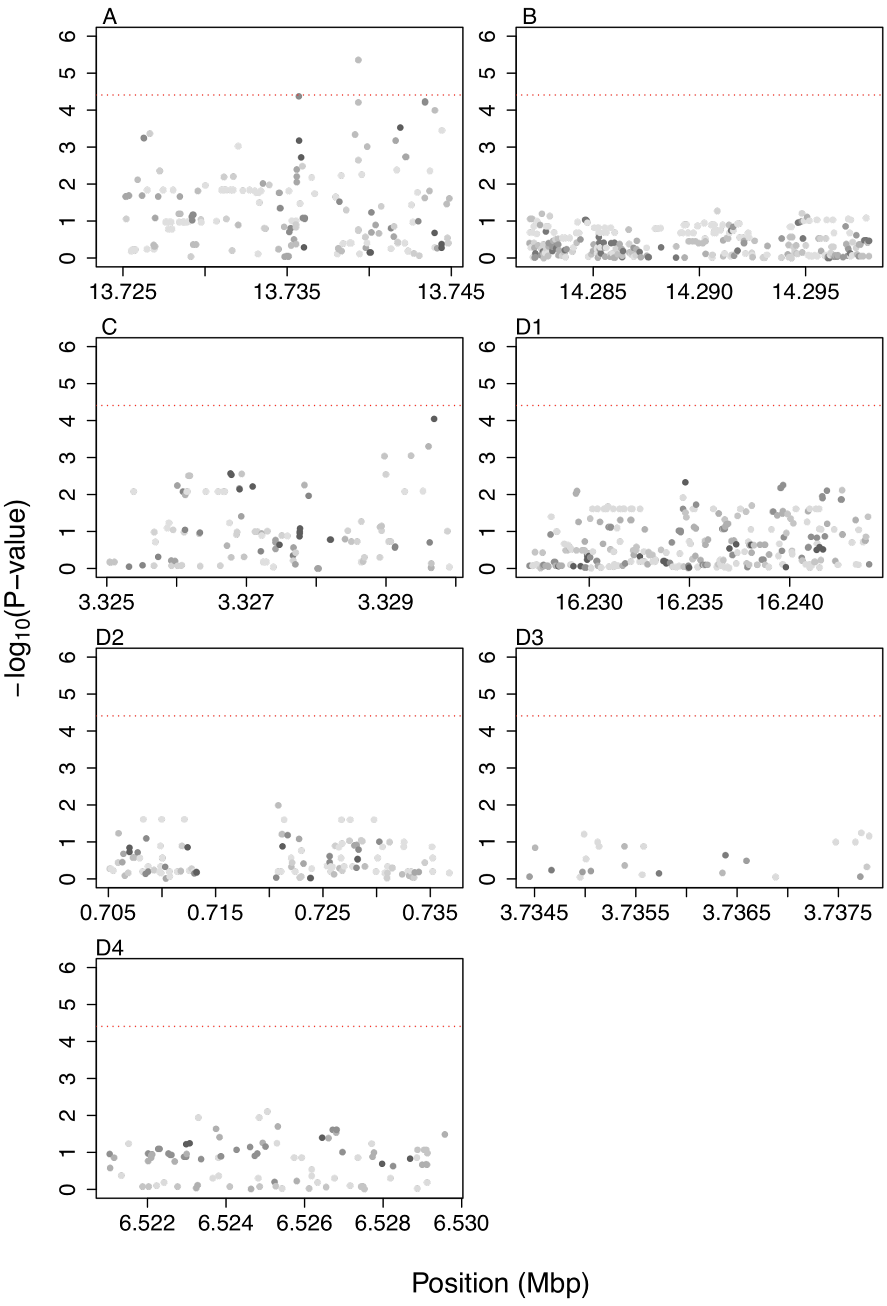

Figure S4 Association scans with MTX toxicity for all SNPs in candidate genes listed in Supplementary Table 3. Red threshold is Bonferroni over all seven candidate gene regions. Symbols are shaded by minor allele frequency in founders represented in RIL panel such that darker circles are more common SNPs. The large gap in D2 is a Gypsy family transposable element present in the reference strain at that position (hence that region in masked in the founders).

a

```
A1      GTTGTTTNNNNNNNNNNNNNNNNNNNNNNNNNNNNNNNNNNNAGCAAGATGATCGAATTTG
A2      GTTGTTTNNNNNNNNNNNNNNNNNNNNNNNNNNNNNNNNGCAAGCAAGATGATCGAATTTG
A3      GTTGTTTAGGTGTAGAGGGGGGGGGGGGGGGTGTTGGTTGCAAGCAAGATGATCGAATTTG
A4      GTTGTTTAGGTGTAGAGGGGGGGGGGGGGGGTGTTGGTTGCAAGCAAGATGATCGAATTTG
A5      GTTGTTTAGGTNNNNNNNNNNNNNNNNNNNNNNNNNNNNNNNNGCAAGATGATCGAATTTG
A6      GTTGTTTAGGTGTAGAGGGGGGGGGGGGGGGTGTTGGTTGCAAGCAAGATGATCGAATTTG
A7      GTTGTTTANNNNNNNNNNNNNNNNNNNNNNNNNNNNNNNGCAAGCAAGATGATCGAATTTG
AB8     GNNNNNNNNNNNNNNNNNNNNNNNNNNNNNNNNNNNNNNGCAAGCAAGATGATCGAATTTG
B1      GTTGNNNNNNNNNNNNNNNNNNNNNNNNNNNNNNNNNNNGCAAGCAAGATGATCGAATTTG
B2      GTTGTTTAGGTGTAGAGGGGGGGGGGGGGGGTGTTGGTTGCAAGCAAGATGATCGAATTTG
B3      GKTGTTTAGGTGTAGAGGGGGGGGGGGGGGGTGTTGGTTGCAAGCAAGATGATCGAATTTG
B4      GTTGNNNNNNNNNNNNNNNNNNNNNNNNNNNNNNNNNNNNNNNGCAAGATGATCGAATTTG
B5      GTTGTTTAGGTGTAGAGGGGGGGGGGGGGGGTGTTGGTTGCAAGCAAGATGATCGAATTTG
B6      GNNNNNNNNNNNNNNNNNNNNNNNNNNGGGGTGTTGGTTGCAAGCAAGATGATCGAATTTG
B7      GTTGTTTAGGTGTAGAGGGGGGGGGGGGGGGGGTTGGTTGCAAGCAAGATGATCGAATTTG
```

b

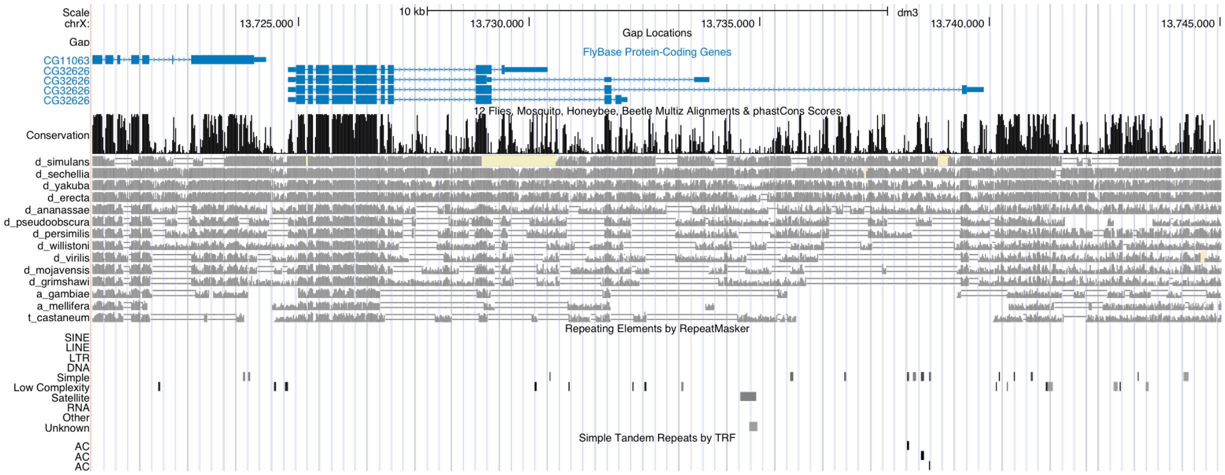

Figure S5 a) Alignment of INDEL polymorphism immediately downstream of exon 1 of CG32626 (the candidate gene for QTL A). The region depicted is the reverse complement of X:13739300-13739360, we reverse complement so the DNA sequence is in the same orientation as transcription. **b)** Santa Cruz Genome Browser screen shot of CG32626 showing four putative alternate first exons.

Table S1 Recovery and "lay-out" Fly Food

Ingredient	Amount[1]
Water	78.4 ml
Agar	0.84 g
Dextrose	6.31 g
Sucrose	3.423 g
K, Na Tartrate	0.96 g
calcium chloride	0.07 g
corn meal	7.6 g
Yeast suspension	
-yeast	3.2 g
-water	20 ml
Propionic acid	1.6 ml
Tegosept	
-tegosept	0.1 g
-ethanol	1 ml

1. For a total of 100 ml

Table S2 Methotrexate *a priori* identified polymorphisms

Human Gene	Polymorphism[1]	Ortholog Type[2]	Fly Gene(s)[2]	References
ABCC2	C24T	Many:1	MRP	Rau, *et al.* 2006
AMPD1	C34T	Many:1	CG32626	Wessels, *et al.* 2006
ATIC	C347G	1:1	CG11089	Wessels, *et al.* 2006
ITPA	C94A	1:1	CG8891	Wessels, *et al.* 2006
MTHFR	C677T	No Ortholog		Vella *et al.* 2011, Kotnik *et al.* 2011,
	A1298C			Kurzawski *et al.* 2007, Seidemann *et al.*
	A80G			2006, Hughes *et al.* 2006
RFC-1	G80A	1:1	Gnf1	Drozdzik *et al.* 2007.
SLCO1B1	3 nsSNPs[3]	No Ortholog		Ramsey, *et al.* 2012
GSTT1/	Complex[4]	Function	GstD1-10[5]	Imanishi, *et al.* 2007
GSTM1/		Function	GstE1-10[5]	
GSTP1				
FPGS	Pathway	1:Many	CG2543	Mikkelsen et al. 2011
	Pathway	1:Many	CG31773	Mikkelsen et al. 2011
GGH	Pathway	1:Many	CG32154	Mikkelsen et al. 2011
	Pathway	1:Many	CG32155	Mikkelsen et al. 2011
TYMS	Pathway	1:1	Ts	Mikkelsen et al. 2011
DHFR	Pathway	Many:1	Dhfr	Mikkelsen et al. 2011
CBS	Pathway	1:1	CG1753	Mikkelsen et al. 2011
MTHFR	Pathway	No Ortholog		Mikkelsen et al. 2011
MTR	Pathway	No Ortholog		Mikkelsen et al. 2011
MTRR	Pathway	1:1	CG14882	Mikkelsen et al. 2011
SHMT1	Pathway	Many:1	CG3011	Mikkelsen et al. 2011
MTHFD1	Pathway	Many:1	pug	Mikkelsen et al. 2011
MTHFS	Pathway	Many:1	CG34424	Mikkelsen et al. 2011
PPAT	Pathway	Many:1	Prat	Mikkelsen et al. 2011
GART	Pathway	1:1	ade3	Mikkelsen et al. 2011
ATIC	Pathway	1:1	CG11089	Mikkelsen et al. 2011
ADA	Pathway	No Ortholog		Mikkelsen et al. 2011

1. Polymorphism refers either to a SNP within a gene (SNP resulting in amino acid substitution given) or “pathway” indicates that the gene is in the MTX cellular pathway based on the literature (but that gene does not harbor a germ-line SNP impacting toxicity).
2. Ortholog types and gene names are represented as on the ensemble.org genome browser (Birney *et al.* 2004)
3. Three non-synonymous SNPs were examined, all had measurable effects
4. A nsSNP in GstP1 may impact toxicity, a presence/absence polymorphism for GstM1 may mediate the impact of RFC1 on toxicity.
5. Gene family. The orthology prediction is based on both human and fly GST gene families having the same apparent biochemical function

Table S3 Candidate genes associated with QTL peaks of Figure 3A and Table 1.

QTL	Gene Name	Chr	Left[1]	Right[1]	nsSNP[2]	SNPs[2]	TEs[2,3]
A	*CG32626*	X	13724	13745	1	233	1{A6}
B	*GstE1-E10*	2R	14282	14298	43	340	2{B2},{A1}
C	*PHGPx*	3L	3325	3330	5	116	0
D1	*CG32154/5*	3L	16227	16244	14	318	1{B3}
D2	*Gnf1*	3R	705	737	8	136	0
D3	*Prat*	3R	3734	3738	1	29	0
D4	*pug*	3R	6521	6530	12	111	0

1. Method for determining Left and Right limits of candidate genes defined in Materials and Methods. Coordinates are given in kilobases.
2. Number of non-synonymous SNPs, other SNPs in the gene region, and transposable elements.
3. All transposable elements were only present in a single founder. Founder line harboring TE in {}.

Table S4   Biallelic SNPs significant a p<0.001 from gene-centric association scans.

QTL	chr	base	$\%V_T$	P	$\%V_G$	MiAC	MaAC
A	X	13726279	3.86%	5.5E-04	6.03%	5	7
A	X	13726288	3.84%	5.7E-04	6.00%	5	7
A	X	13726642	4.01%	4.3E-04	6.26%	2	10
A	X	13732029	3.54%	9.3E-04	5.54%	1	11
A	X	13735714	5.38%	4.2E-05	8.40%	7	5
A	X	13735738	3.76%	6.5E-04	5.87%	6	6
A	X	13739146	3.98%	4.5E-04	6.21%	3	9
A	X	13739343	5.15%	6.1E-05	8.05%	3	9
A	X	13739344	6.72%	4.3E-06	10.50%	3	9
A	X	13739888	3.53%	9.5E-04	5.52%	3	9
A	X	13741615	3.75%	6.6E-04	5.85%	4	8
A	X	13741897	4.22%	3.0E-04	6.60%	6	6
A	X	13743413	5.18%	5.8E-05	8.10%	5	7
A	X	13743414	5.15%	6.2E-05	8.05%	5	7
A	X	13744000	4.87%	1.0E-04	7.60%	3	9
A	X	13744431	4.13%	3.5E-04	6.45%	1	11
C	3L	3328980	3.57%	9.0E-04	5.58%	3	11
C	3L	3329361	3.57%	8.9E-04	5.58%	3	11
C	3L	3329612	3.92%	5.0E-04	6.12%	4	10
C	3L	3329692	4.94%	8.9E-05	7.71%	7	7

Note: QTL corresponds to QTL in Supplementary Table 3, chr=chromosome, base=base position in chromosome, $\%V_T$=percent of total variation explained by SNP, P=p-value, $\%V_G$=percent of genetic variation explained by SNP, MiAC= Minor Allele Count = Number of founder chromosomes having minor allele represented in panel at this position, MaAC=Major Allele Count. Shaded rows are most significant SNP in each gene (SNP under QTL A is Bonferroni significant over all 7 candidate gene regions, SNP under QTL C is not-Bonferroni significant). The other 5 genes had no SNPs significant at p<0.001.

References appearing only in supporting information

Drozdzik M., Rudas T., Pawlik A., Gornik W., Kurzawski M., et al., 2007 Reduced folate carrier-1 80G>A polymorphism affects methotrexate treatment outcome in rheumatoid arthritis. Pharmacogenomics J. 7(6): 404-7.

Hughes L. B., Beasley T. M., Patel H., Tiwari H. K., Morgan S. L., et al., 2006 Racial or ethnic differences in allele frequencies of single-nucleotide polymorphisms in the methylenetetrahydrofolate reductase gene and their influence on response to methotrexate in rheumatoid arthritis. Ann. Rheum. Dis. 65(9): 1213-8.

Kotnik B., Grabnar I,. Grabar P., Dolzan V., Jazbec J., 2011 Association of gentic polymorphism in the folate metabolic pathway with methotrexate pharmacokinetics and toxicity in chilhood acute lymphoblastic leukaemia and malignant lymphoma. Eur. J. Clin. Pharmacol. 67(10): 993-1006.

Kurzawski M., Pawlik A., Safranow K., Herczynska M., Drozdzik M., 2007 677C>T and 1298A>C MTHFR polymorphisms affect methotrexate treatment outcome in rheumatoid arthritis. Pharmacogenomics 8(11): 1551-9.

Ramsey L. B., Panetta J. C., Smith C., Yang W., Fan Y., Winick N. J., Martin P. L., Cheng C., Devidas M., Pui C. H., Evans W. E., Hunger S. P., Loh M., Relling M. V., 2012 Genome-wide study of methotrexate clearance replicates SLCO1B1. Blood 121(6): 898-904.

Rau T., Erney B., Gores R., Eschenhagen T., Beck J., Langer T., 2006 High-dose methotrexate in pediatric acute lymphoblastic leukemia: impact of ABCC2 polymorphisms on plasma concentrations. Clin Pharmacol Ther 80(5): 468-76.
