# Supplementary material for: The Genetic Architecture of Methotrexate Toxicity Is Similar in Drosophila melanogaster and Humans
Source: G3 (Bethesda). 2013 Aug 1;3(8):1301–10. doi: 10.1534/g3.113.006619 (PMC3737169; doi:10.1534/g3.113.006619)
Supplement: Supporting Information [file supp_g3.113.006619_FigureS1.pdf]

a

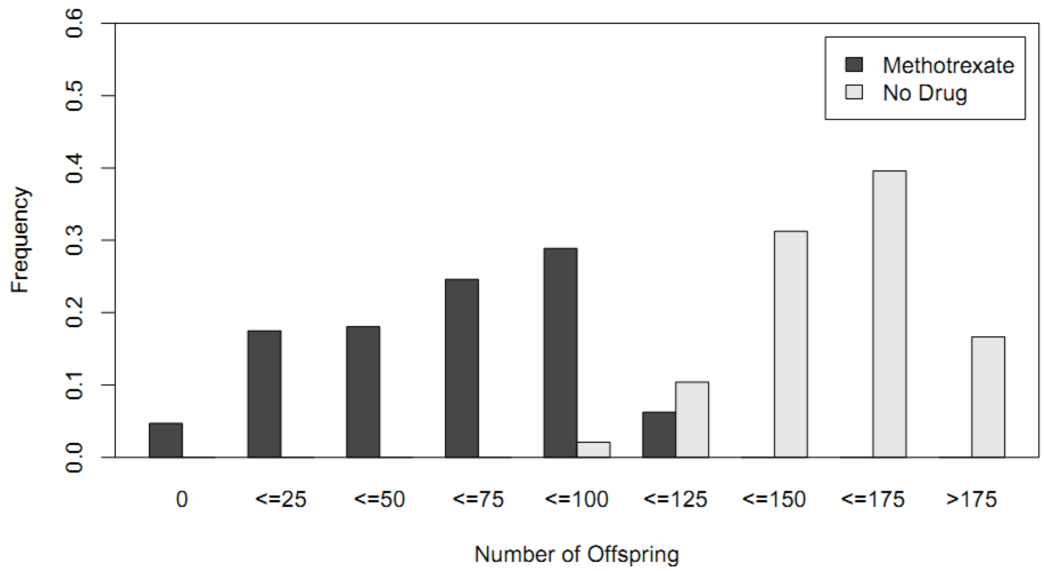

b

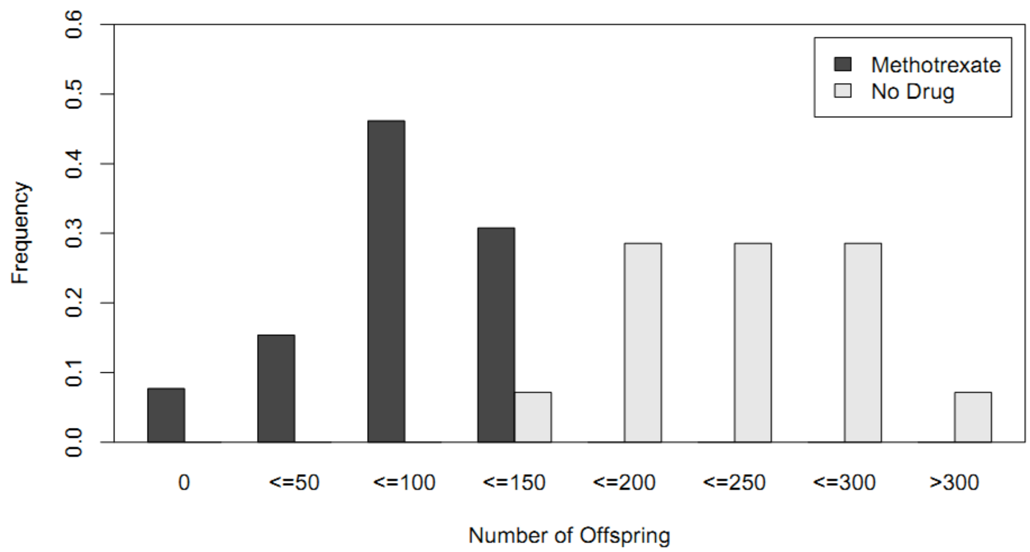

Figure S1 Saturation and total percent knockdown. **a)** Saturation: A “no drug” treatment was carried out in fly condos in order to assess the saturation point of the condo wells. A total of 48 wells were filled with 5 pairs of flies each, and allowed to lay eggs for 2 days. In two weeks, the number of offspring was counted and compared to the number of offspring produced by all RIL crosses of the MTX-treated assay. **b)** Percent knock-down: 16 genotypes were treated using our standard dosing protocol in condos. Three treated sets of 3 males and 3 females per genotype were then allowed to lay eggs for 48 hours in condos. One mock treated set of 3 males and 3 females per genotype were also allowed to lay eggs in bottles for 48 hours. We compare the mean of each treated genotype (dark grey) to the same genotype from the mock-treated fecundity assayed in half-pint round-bottom glass stock bottles (light grey).
