# Supplementary material for: The Genetic Architecture of Methotrexate Toxicity Is Similar in Drosophila melanogaster and Humans
Source: G3 (Bethesda). 2013 Aug 1;3(8):1301–10. doi: 10.1534/g3.113.006619 (PMC3737169; doi:10.1534/g3.113.006619)
Supplement: Supporting Information [file supp_g3.113.006619_SupportingReferences.pdf]

References appearing only in supporting information

Drozdzik M., Rudas T., Pawlik A., Gornik W., Kurzawski M., et al., 2007 Reduced folate carrier-1 80G>A polymorphism affects methotrexate treatment outcome in rheumatoid arthritis. Pharmacogenomics J. 7(6): 404-7.

Hughes L. B., Beasley T. M., Patel H., Tiwari H. K., Morgan S. L., et al., 2006 Racial or ethnic differences in allele frequencies of single-nucleotide polymorphisms in the methylenetetrahydrofolate reductase gene and their influence on response to methotrexate in rheumatoid arthritis. Ann. Rheum. Dis. 65(9): 1213-8.

Kotnik B., Grabnar I,. Grabar P., Dolzan V., Jazbec J., 2011 Association of gentic polymorphism in the folate metabolic pathway with methotrexate pharmacokinetics and toxicity in chilhood acute lymphoblastic leukaemia and malignant lymphoma. Eur. J. Clin. Pharmacol. 67(10): 993-1006.

Kurzawski M., Pawlik A., Safranow K., Herczynska M., Drozdzik M., 2007 677C>T and 1298A>C MTHFR polymorphisms affect methotrexate treatment outcome in rheumatoid arthritis. Pharmacogenomics 8(11): 1551-9.

Ramsey L. B., Panetta J. C., Smith C., Yang W., Fan Y., Winick N. J., Martin P. L., Cheng C., Devidas M., Pui C. H., Evans W. E., Hunger S. P., Loh M., Relling M. V., 2012 Genome-wide study of methotrexate clearance replicates SLCO1B1. Blood 121(6): 898-904.

Rau T., Erney B., Gores R., Eschenhagen T., Beck J., Langer T., 2006 High-dose methotrexate in pediatric acute lymphoblastic leukemia: impact of ABCC2 polymorphisms on plasma concentrations. Clin Pharmacol Ther 80(5): 468-76.
