# Supplementary material for: The Genetic Architecture of Methotrexate Toxicity Is Similar in Drosophila melanogaster and Humans
Source: G3 (Bethesda). 2013 Aug 1;3(8):1301–10. doi: 10.1534/g3.113.006619 (PMC3737169; doi:10.1534/g3.113.006619)
Supplement: Supporting Information [file supp_g3.113.006619_FigureS2.pdf]

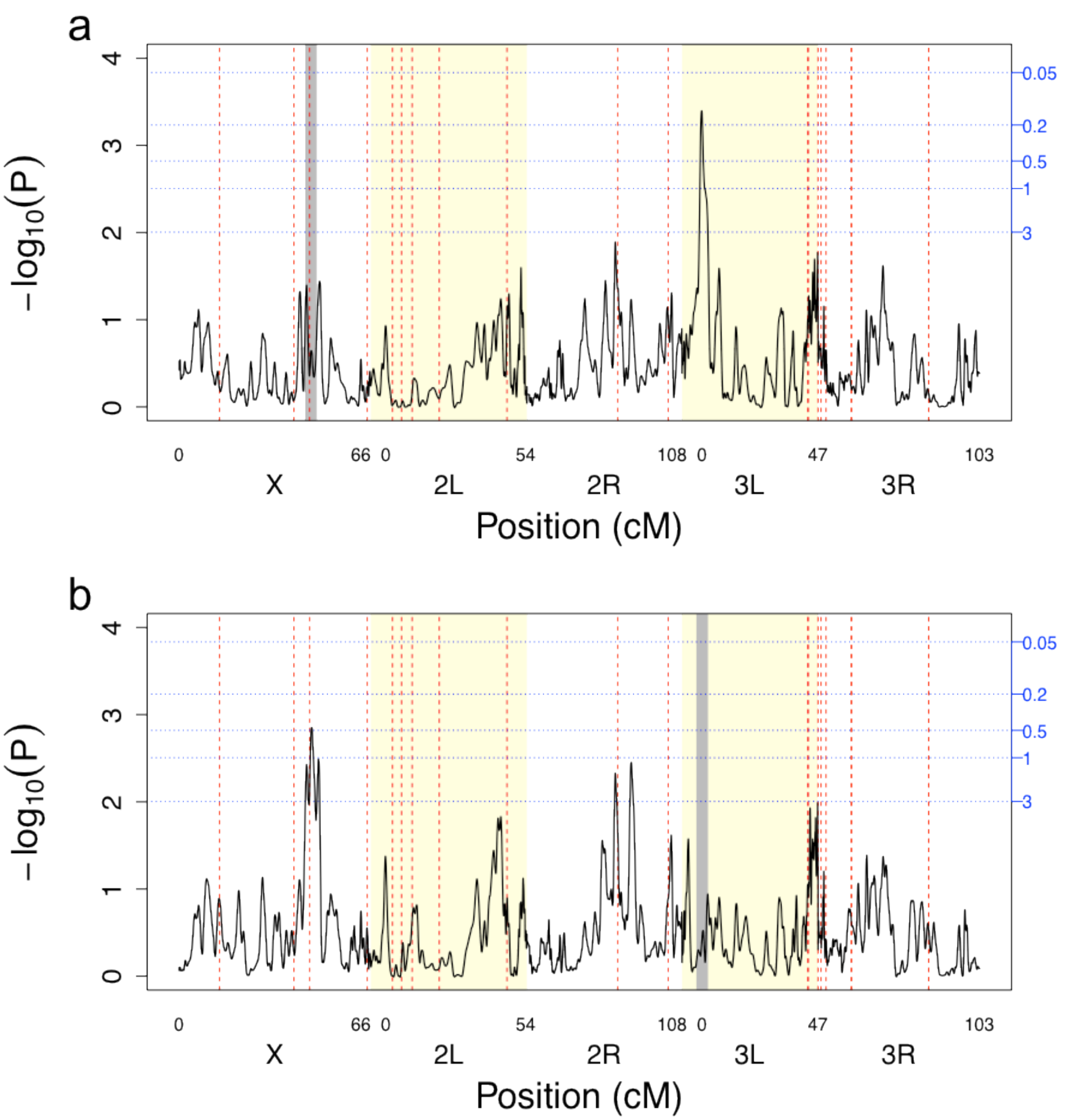

Figure S2 Residual methotrexate toxicity genome scans adjusting for **a)** QTL A and **b)** QTL C. The grey vertical bar indicates the region within 2cM of the QTL corrected for and therefore where results should be interpreted with caution. Horizontal blue dotted lines indicate thresholds for various false positive rates (number of expected peaks per genome scan) noted on the right y-axis. Vertical dashed red lines indicate the location of fly orthologs of previously identified human candidate genes for methotrexate toxicity.
