# Supplementary material for: The Genetic Architecture of Methotrexate Toxicity Is Similar in Drosophila melanogaster and Humans
Source: G3 (Bethesda). 2013 Aug 1;3(8):1301–10. doi: 10.1534/g3.113.006619 (PMC3737169; doi:10.1534/g3.113.006619)
Supplement: Supporting Information [file supp_g3.113.006619_FigureS3.pdf]

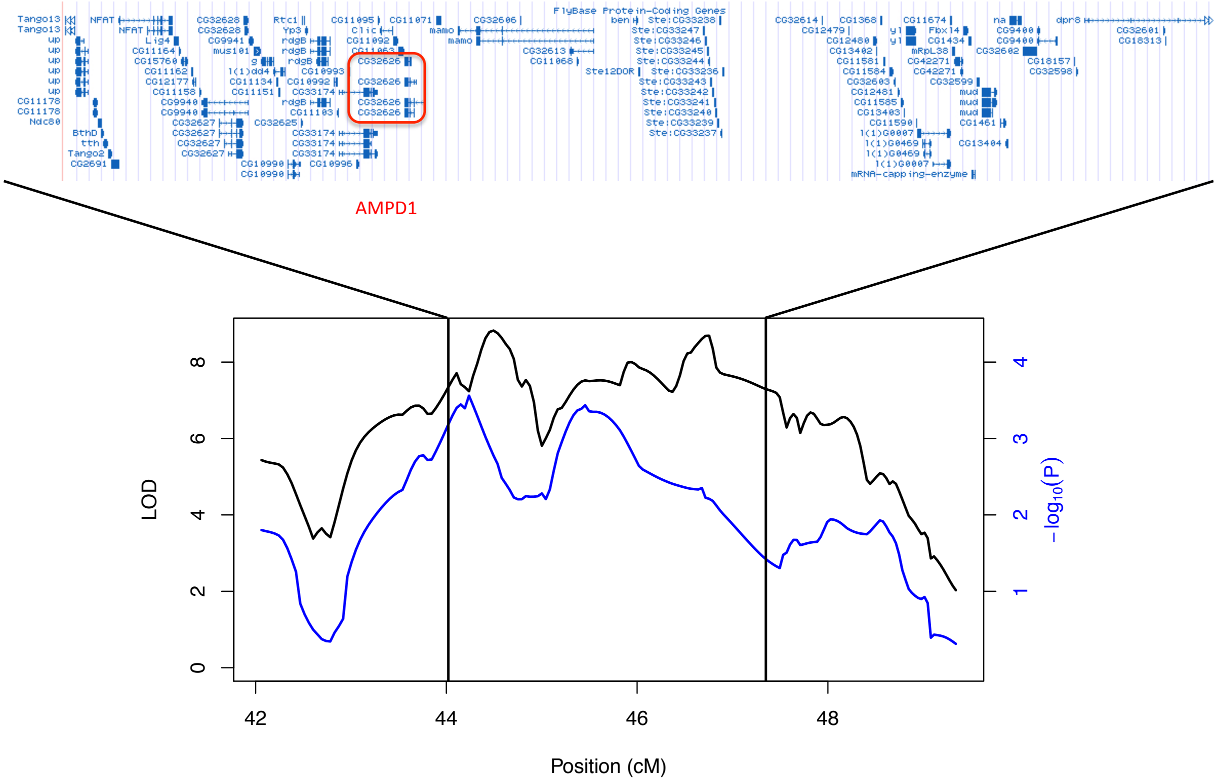
FlyBase Protein-Coding Genes
AMPD1
LOD
$-\log_{10}(P)$
Position (cM)
42
44
46
48
0
2
4
6
8
1
2
3
4

QTL A

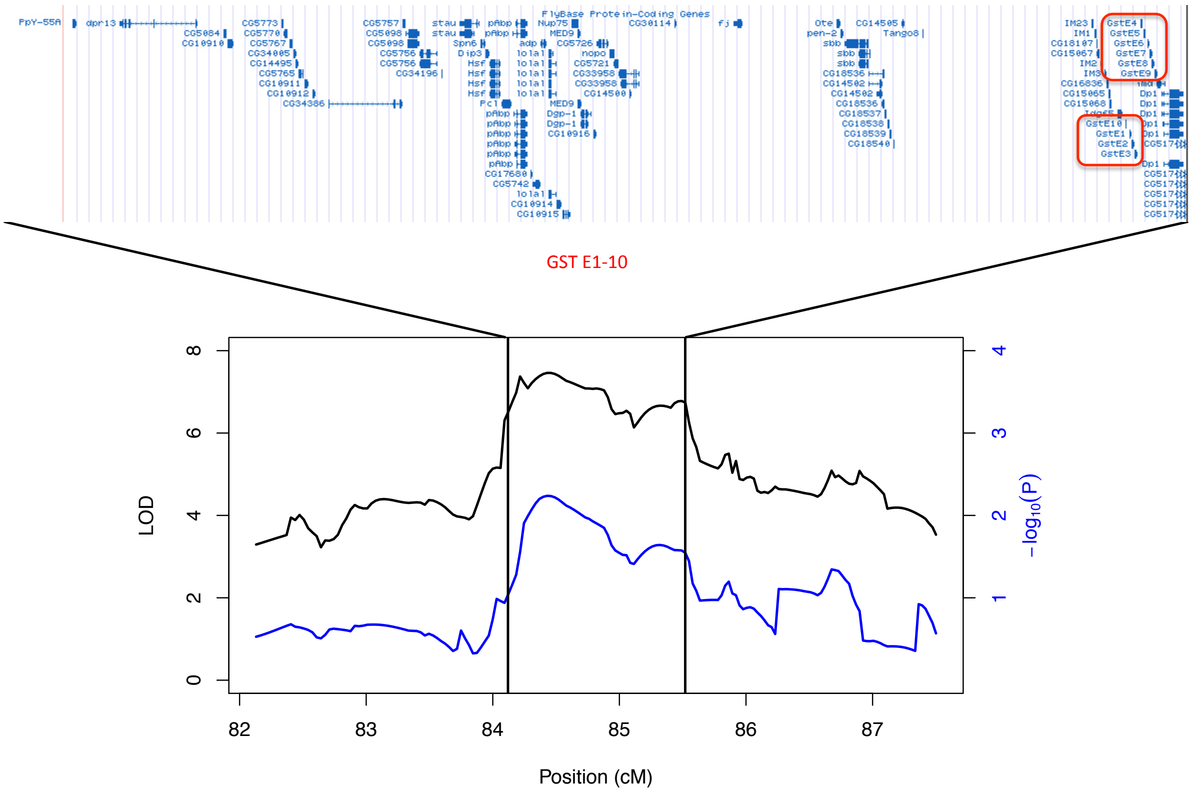
FlyBase Protein-Coding Genes
GST E1-10
LOD
$-\log_{10}(P)$
Position (cM)

QTL B

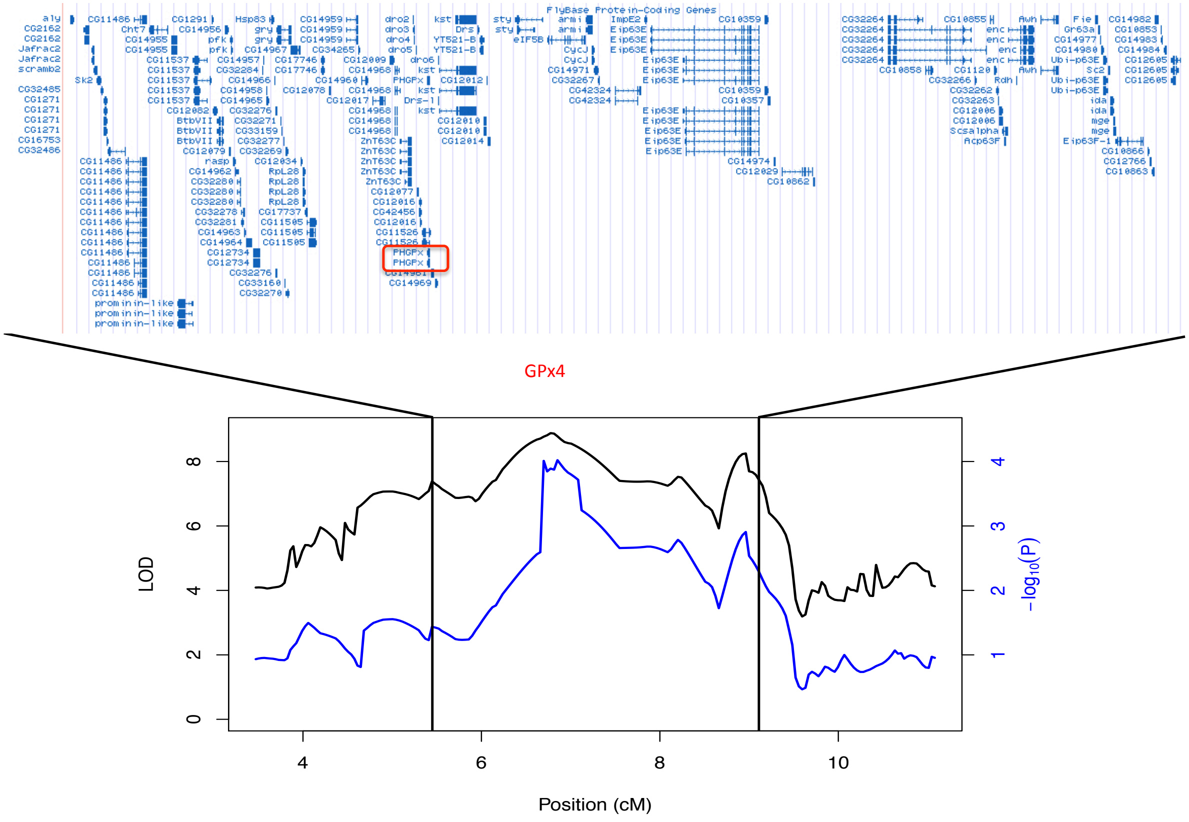

QTL C

Figure S3 Santa Cruz Genome Browser for Bayesian Credible Intervals (Table1) for QTLs A-C. QTL D spans a centromere so is poorly resolved. For QTLs A&B the Drosophila orthologs of a priori human candidate genes is circled in red with the associated human gene name also in red. For QTL C the novel candidate gene is circled in red, with the associated human genes name also given.
