# Supplementary material for: The Genetic Architecture of Methotrexate Toxicity Is Similar in Drosophila melanogaster and Humans
Source: G3 (Bethesda). 2013 Aug 1;3(8):1301–10. doi: 10.1534/g3.113.006619 (PMC3737169; doi:10.1534/g3.113.006619)
Supplement: Supporting Information [file supp_g3.113.006619_FigureS4.pdf]

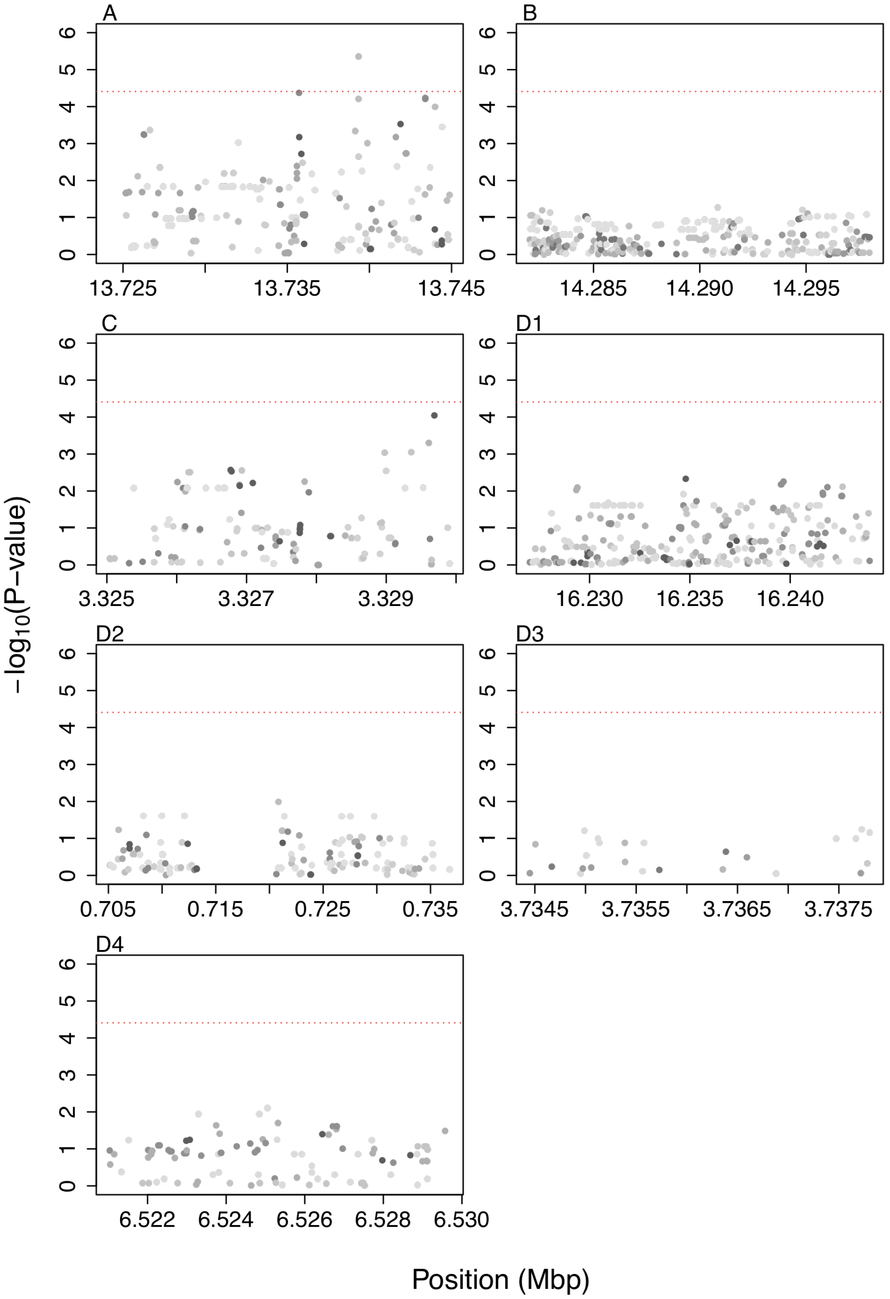

Figure S4 Association scans with MTX toxicity for all SNPs in candidate genes listed in Supplementary Table 3. Red threshold is Bonferroni over all seven candidate gene regions. Symbols are shaded by minor allele frequency in founders represented in RIL panel such that darker circles are more common SNPs. The large gap in D2 is a Gypsy family transposable element present in the reference strain at that position (hence that region in masked in the founders).
