# Supplementary material for: The Genetic Architecture of Methotrexate Toxicity Is Similar in Drosophila melanogaster and Humans
Source: G3 (Bethesda). 2013 Aug 1;3(8):1301–10. doi: 10.1534/g3.113.006619 (PMC3737169; doi:10.1534/g3.113.006619)
Supplement: Supporting Information [file supp_g3.113.006619_FigureS5.pdf]

a

```
A1      GTTGTTTNNNNNNNNNNNNNNNNNNNNNNNNNNNNNNNNNNNAGCAAGATGATCGAATTTG
A2      GTTGTTTNNNNNNNNNNNNNNNNNNNNNNNNNNNNNNNNGCAAGCAAGATGATCGAATTTG
A3      GTTGTTTAGGTGTAGAGGGGGGGGGGGGGGGTGTTGGTTGCAAGCAAGATGATCGAATTTG
A4      GTTGTTTAGGTGTAGAGGGGGGGGGGGGGGGTGTTGGTTGCAAGCAAGATGATCGAATTTG
A5      GTTGTTTAGGTNNNNNNNNNNNNNNNNNNNNNNNNNNNNNNNNGCAAGATGATCGAATTTG
A6      GTTGTTTAGGTGTAGAGGGGGGGGGGGGGGGTGTTGGTTGCAAGCAAGATGATCGAATTTG
A7      GTTGTTTANNNNNNNNNNNNNNNNNNNNNNNNNNNNNNNGCAAGCAAGATGATCGAATTTG
AB8     GNNNNNNNNNNNNNNNNNNNNNNNNNNNNNNNNNNNNNNGCAAGCAAGATGATCGAATTTG
B1      GTTGNNNNNNNNNNNNNNNNNNNNNNNNNNNNNNNNNNNGCAAGCAAGATGATCGAATTTG
B2      GTTGTTTAGGTGTAGAGGGGGGGGGGGGGGGTGTTGGTTGCAAGCAAGATGATCGAATTTG
B3      GKTGTTTAGGTGTAGAGGGGGGGGGGGGGGGTGTTGGTTGCAAGCAAGATGATCGAATTTG
B4      GTTGNNNNNNNNNNNNNNNNNNNNNNNNNNNNNNNNNNNNNNNGCAAGATGATCGAATTTG
B5      GTTGTTTAGGTGTAGAGGGGGGGGGGGGGGGTGTTGGTTGCAAGCAAGATGATCGAATTTG
B6      GNNNNNNNNNNNNNNNNNNNNNNNNNNNGGGTGTTGGTTGCAAGCAAGATGATCGAATTTG
B7      GTTGTTTAGGTGTAGAGGGGGGGGGGGGGGGGGTTGGTTGCAAGCAAGATGATCGAATTTG
```

b

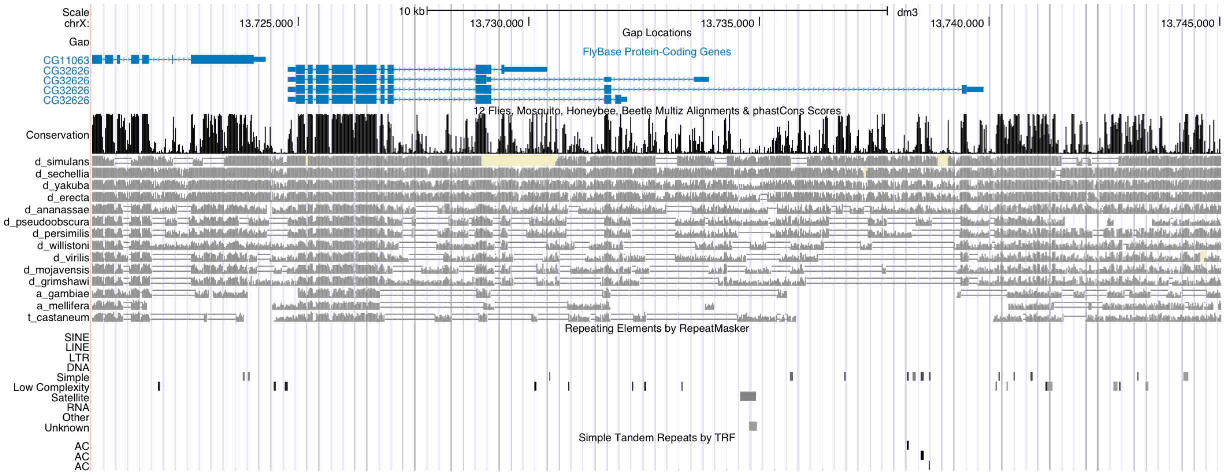

Figure S5 a) Alignment of INDEL polymorphism immediately downstream of exon 1 of CG32626 (the candidate gene for QTL A). The region depicted is the reverse complement of X:13739300-13739360, we reverse complement so the DNA sequence is in the same orientation as transcription. **b)** Santa Cruz Genome Browser screen shot of CG32626 showing four putative alternate first exons.
