# Supplementary material for: The Genetic Architecture of Methotrexate Toxicity Is Similar in Drosophila melanogaster and Humans
Source: G3 (Bethesda). 2013 Aug 1;3(8):1301–10. doi: 10.1534/g3.113.006619 (PMC3737169; doi:10.1534/g3.113.006619)
Supplement: Supporting Information [file supp_g3.113.006619_TableS1.pdf]

Table S1 Recovery and "lay-out" Fly Food

Ingredient	Amount[1]
Water	78.4 ml
Agar	0.84 g
Dextrose	6.31 g
Sucrose	3.423 g
K, Na Tartrate	0.96 g
calcium chloride	0.07 g
corn meal	7.6 g
Yeast suspension	
-yeast	3.2 g
-water	20 ml
Propionic acid	1.6 ml
Tegosept	
-tegosept	0.1 g
-ethanol	1 ml

1. For a total of 100 ml
