# Supplementary material for: The Genetic Architecture of Methotrexate Toxicity Is Similar in Drosophila melanogaster and Humans
Source: G3 (Bethesda). 2013 Aug 1;3(8):1301–10. doi: 10.1534/g3.113.006619 (PMC3737169; doi:10.1534/g3.113.006619)
Supplement: Supporting Information [file supp_g3.113.006619_TableS2.pdf]

Table S2 Methotrexate *a priori* identified polymorphisms

Human Gene	Polymorphism[1]	Ortholog Type[2]	Fly Gene(s)[2]	References
ABCC2	C24T	Many:1	MRP	Rau, *et al.* 2006
AMPD1	C34T	Many:1	CG32626	Wessels, *et al.* 2006
ATIC	C347G	1:1	CG11089	Wessels, *et al.* 2006
ITPA	C94A	1:1	CG8891	Wessels, *et al.* 2006
MTHFR	C677T	No Ortholog		Vella *et al.* 2011, Kotnik *et al.* 2011,
	A1298C			Kurzawski *et al.* 2007, Seidemann *et al.*
	A80G			2006, Hughes *et al.* 2006
RFC-1	G80A	1:1	Gnf1	Drozdzik *et al.* 2007.
SLCO1B1	3 nsSNPs[3]	No Ortholog		Ramsey, *et al.* 2012
GSTT1/	Complex[4]	Function	GstD1-10[5]	Imanishi, *et al.* 2007
GSTM1/		Function	GstE1-10[5]	
GSTP1				
FPGS	Pathway	1:Many	CG2543	Mikkelsen et al. 2011
	Pathway	1:Many	CG31773	Mikkelsen et al. 2011
GGH	Pathway	1:Many	CG32154	Mikkelsen et al. 2011
	Pathway	1:Many	CG32155	Mikkelsen et al. 2011
TYMS	Pathway	1:1	Ts	Mikkelsen et al. 2011
DHFR	Pathway	Many:1	Dhfr	Mikkelsen et al. 2011
CBS	Pathway	1:1	CG1753	Mikkelsen et al. 2011
MTHFR	Pathway	No Ortholog		Mikkelsen et al. 2011
MTR	Pathway	No Ortholog		Mikkelsen et al. 2011
MTRR	Pathway	1:1	CG14882	Mikkelsen et al. 2011
SHMT1	Pathway	Many:1	CG3011	Mikkelsen et al. 2011
MTHFD1	Pathway	Many:1	pug	Mikkelsen et al. 2011
MTHFS	Pathway	Many:1	CG34424	Mikkelsen et al. 2011
PPAT	Pathway	Many:1	Prat	Mikkelsen et al. 2011
GART	Pathway	1:1	ade3	Mikkelsen et al. 2011
ATIC	Pathway	1:1	CG11089	Mikkelsen et al. 2011
ADA	Pathway	No Ortholog		Mikkelsen et al. 2011

1. Polymorphism refers either to a SNP within a gene (SNP resulting in amino acid substitution given) or "pathway" indicates that the gene is in the MTX cellular pathway based on the literature (but that gene does not harbor a germ-line SNP impacting toxicity).
2. Ortholog types and gene names are represented as on the ensemble.org genome browser (Birney *et al.* 2004)
3. Three non-synonymous SNPs were examined, all had measurable effects
4. A nsSNP in GstP1 may impact toxicity, a presence/absence polymorphism for GstM1 may mediate the impact of RFC1 on toxicity.
5. Gene family. The orthology prediction is based on both human and fly GST gene families having the same apparent biochemical function
