# Supplementary material for: The Genetic Architecture of Methotrexate Toxicity Is Similar in Drosophila melanogaster and Humans
Source: G3 (Bethesda). 2013 Aug 1;3(8):1301–10. doi: 10.1534/g3.113.006619 (PMC3737169; doi:10.1534/g3.113.006619)
Supplement: Supporting Information [file supp_g3.113.006619_TableS3.pdf]

Table S3 Candidate genes associated with QTL peaks of Figure 3A and Table 1.

QTL	Gene Name	Chr	Left[1]	Right[1]	nsSNP[2]	SNPs[2]	TEs[2,3]
A	*CG32626*	X	13724	13745	1	233	1{A6}
B	*GstE1-E10*	2R	14282	14298	43	340	2{B2},{A1}
C	*PHGPx*	3L	3325	3330	5	116	0
D1	*CG32154/5*	3L	16227	16244	14	318	1{B3}
D2	*Gnf1*	3R	705	737	8	136	0
D3	*Prat*	3R	3734	3738	1	29	0
D4	*pug*	3R	6521	6530	12	111	0

1. Method for determining Left and Right limits of candidate genes defined in Materials and Methods. Coordinates are given in kilobases.
2. Number of non-synonymous SNPs, other SNPs in the gene region, and transposable elements.
3. All transposable elements were only present in a single founder. Founder line harboring TE in {}.
