# Supplementary material for: The Genetic Architecture of Methotrexate Toxicity Is Similar in Drosophila melanogaster and Humans
Source: G3 (Bethesda). 2013 Aug 1;3(8):1301–10. doi: 10.1534/g3.113.006619 (PMC3737169; doi:10.1534/g3.113.006619)
Supplement: Supporting Information [file supp_g3.113.006619_TableS4.pdf]

Table S4 Biallelic SNPs significant a p<0.001 from gene-centric association scans.

QTL	chr	base	$\%V_T$	P	$\%V_G$	MiAC	MaAC
A	X	13726279	3.86%	5.5E-04	6.03%	5	7
A	X	13726288	3.84%	5.7E-04	6.00%	5	7
A	X	13726642	4.01%	4.3E-04	6.26%	2	10
A	X	13732029	3.54%	9.3E-04	5.54%	1	11
A	X	13735714	5.38%	4.2E-05	8.40%	7	5
A	X	13735738	3.76%	6.5E-04	5.87%	6	6
A	X	13739146	3.98%	4.5E-04	6.21%	3	9
A	X	13739343	5.15%	6.1E-05	8.05%	3	9
A	X	13739344	6.72%	4.3E-06	10.50%	3	9
A	X	13739888	3.53%	9.5E-04	5.52%	3	9
A	X	13741615	3.75%	6.6E-04	5.85%	4	8
A	X	13741897	4.22%	3.0E-04	6.60%	6	6
A	X	13743413	5.18%	5.8E-05	8.10%	5	7
A	X	13743414	5.15%	6.2E-05	8.05%	5	7
A	X	13744000	4.87%	1.0E-04	7.60%	3	9
A	X	13744431	4.13%	3.5E-04	6.45%	1	11
C	3L	3328980	3.57%	9.0E-04	5.58%	3	11
C	3L	3329361	3.57%	8.9E-04	5.58%	3	11
C	3L	3329612	3.92%	5.0E-04	6.12%	4	10
C	3L	3329692	4.94%	8.9E-05	7.71%	7	7

Note: QTL corresponds to QTL in Supplementary Table 3, chr=chromosome, base=base position in chromosome, $\%V_T$=percent of total variation explained by SNP, P=p-value, $\%V_G$=percent of genetic variation explained by SNP, MiAC= Minor Allele Count = Number of founder chromosomes having minor allele represented in panel at this position, MaAC=Major Allele Count. Shaded rows are most significant SNP in each gene (SNP under QTL A is Bonferroni significant over all 7 candidate gene regions, SNP under QTL C is not-Bonferroni significant). The other 5 genes had no SNPs significant at p<0.001.
